# Supplementary material for: Combination therapies induce cancer cell death through the integrated stress response and disturbed pyrimidine metabolism
Source: EMBO Mol Med. 2021 Mar 5;13(4):e12461. doi: 10.15252/emmm.202012461 (PMC8033521; doi:10.15252/emmm.202012461)
Supplement: Supplementary file 1 — Appendix [file EMMM-13-e12461-s003.pdf]

APPENDIX FIGURES AND TABLE

Table of content

Appendix Figure S1:	Dose-response analyses for the individual drugs
Appendix Figure S2:	GO gene set enrichment analysis (GSEA) of the transcriptome data
Appendix Figure S3:	Heat map of differentially expressed genes (DEGs) from the transcriptomic analysis
Appendix Figures S4A, B:	Immunoblots for ISR proteins (Chop, ATF4, p-/t-eIF2a)
Appendix Figure S5:	WikiPathways gene set enrichment analysis (GSEA) of the transcriptome data
Appendix Figures S6:	Heat map of CLEAR network gene expression from the transcriptome analysis
Appendix Figure S7:	Volcano blot of DEGs from the transcriptome analysis (UPP1 highlighted)
Appendix Figure S8:	Induction of UPP1 protein levels upon drug treatments
Appendix Table S1:	Statistical tests and exact p-values

Appendix Figure S1: Dose-response analyses for the individual drugs

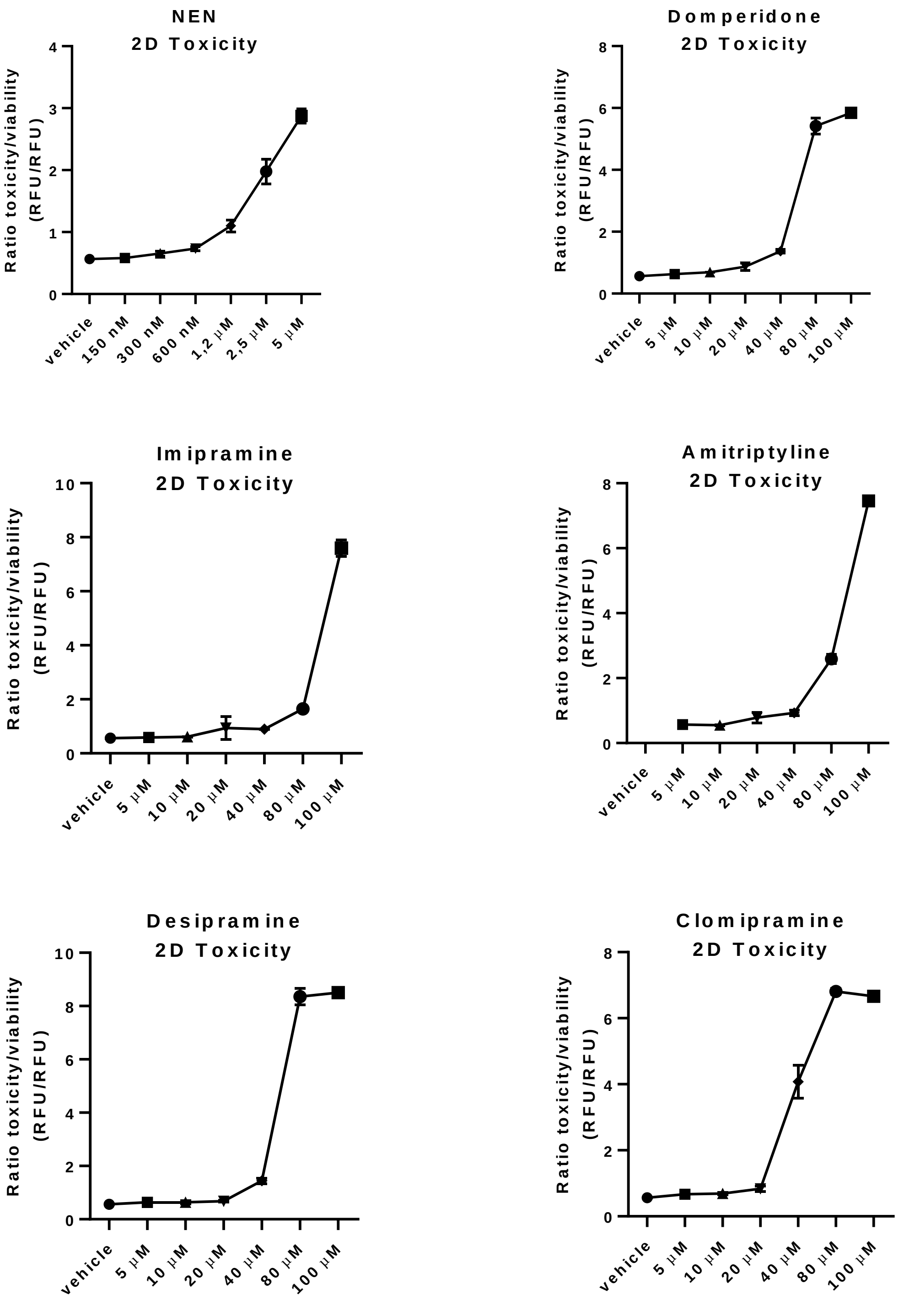

Legend: HCT116 cells were treated with increasing concentrations of NEN and the individual drugs as indicated. Graphs shows the ratio of toxicity and viability measured after a 24h treatment (N=3). The concentrations used for determination of cytotoxicity effects throughout the study were NEN 1.2 µM; Domperidone (Domp), Imipramine (Imi), Desipramine (Desi) and Amitriptyline (Ami), each 30 µM; Clomipramine (Clomi) 20 µM.

Appendix Figure S2: GO gene set enrichment analysis (GSEA) of transcriptome data

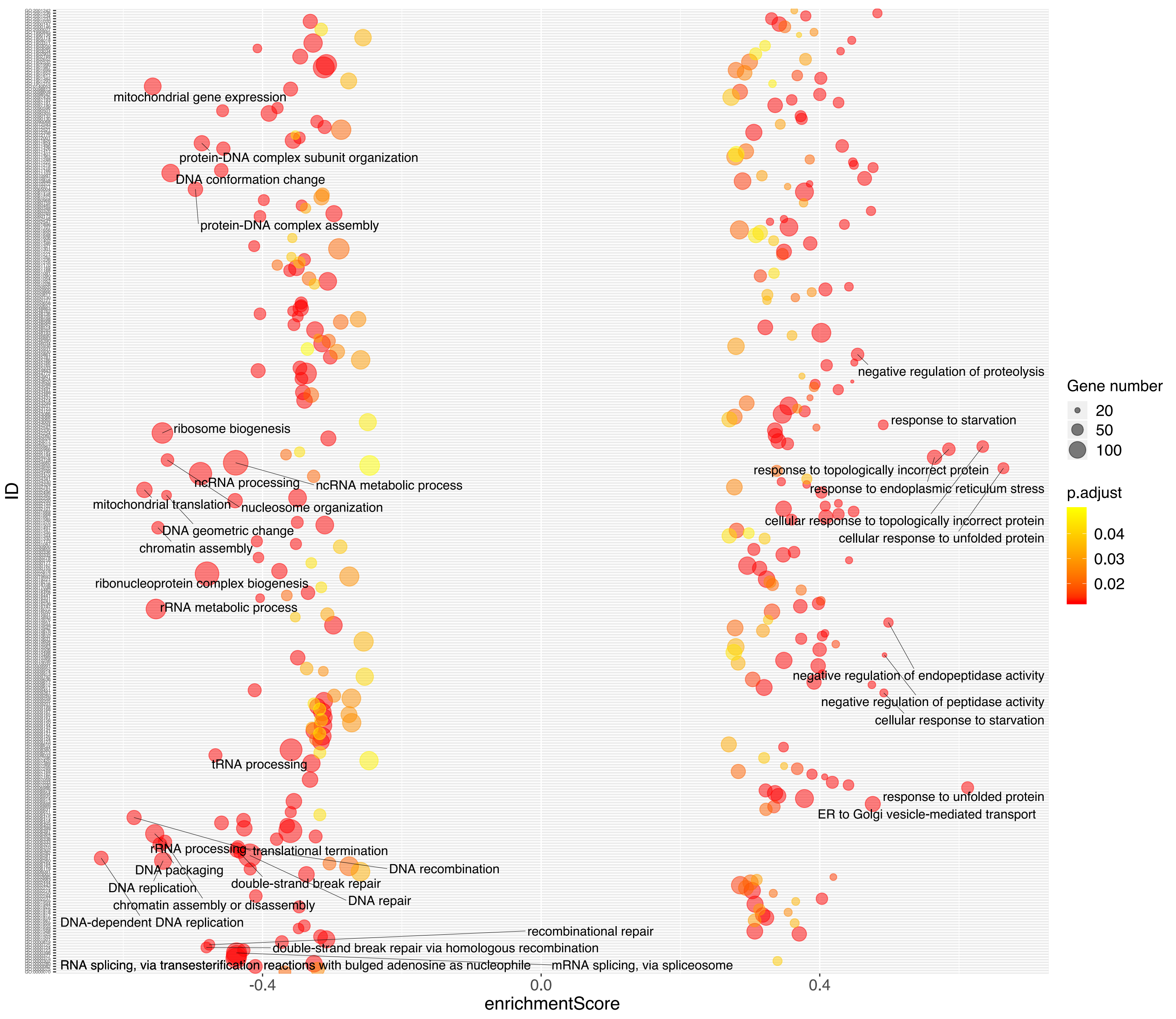

Legend: GO gene set enrichment analysis (GSEA) of microarray data from HCT116 cells upon double treatment with NEN (1.2µM) and Domperidone (30µM) for 16 hours vs. vehicle control treatment (N=3).

Appendix Figure S3: Heat map of differentially expressed genes (DEGs) from the transcriptome analysis

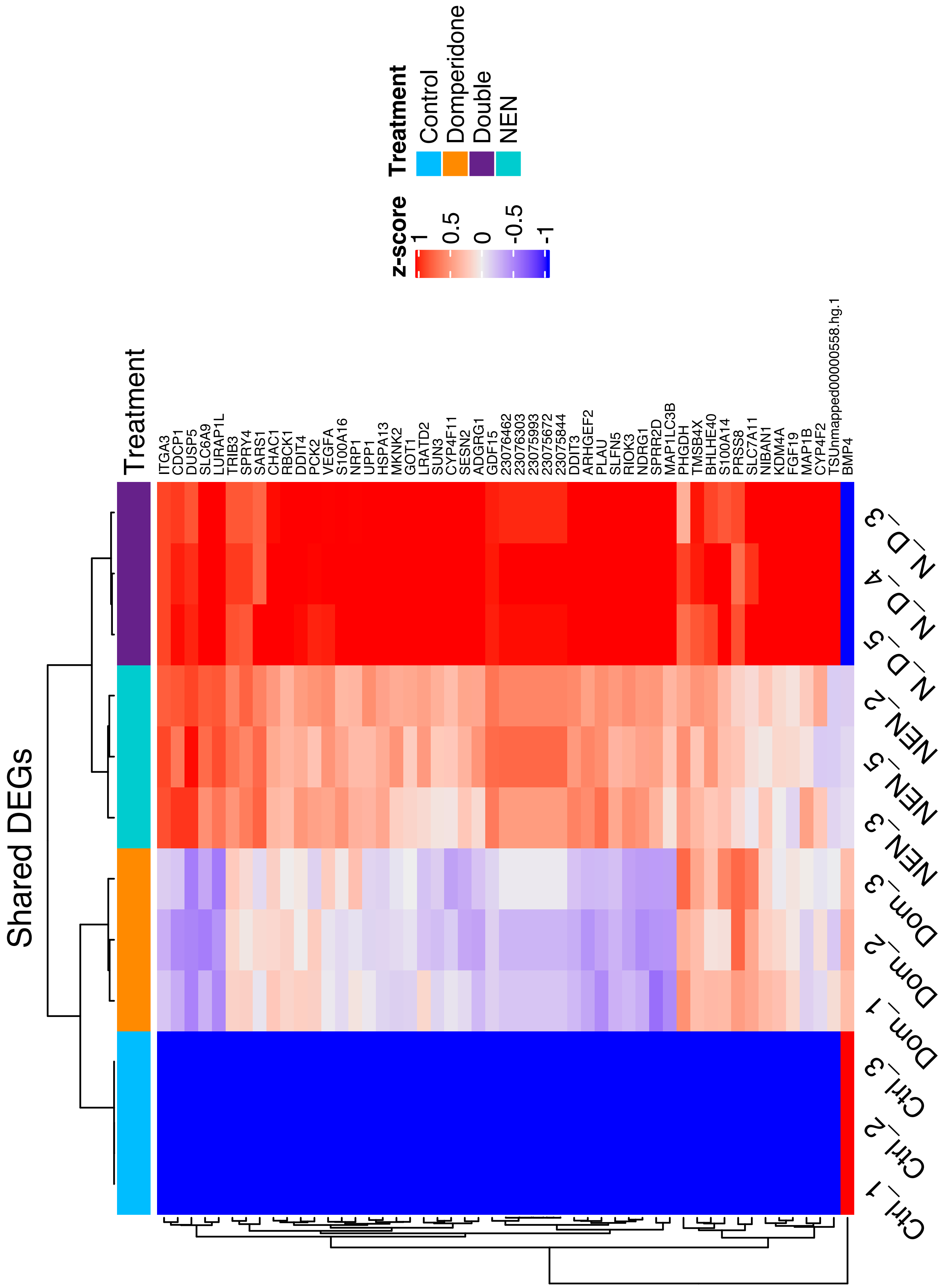

Legend: Differentially expressed genes determined from the microarray analysis of HCT116 cells upon treatment with vehicle control (Ctrl), NEN (1.2μM), Domperidone (Dom, 30μM) or double treatment with NEN and Domperidone for 16 hours. Genes showing synergistic induction upon double treatment in comparison to single on control treatment are shown.

Appendix Figure S4A: Induction of ISR proteins upon drug treatments

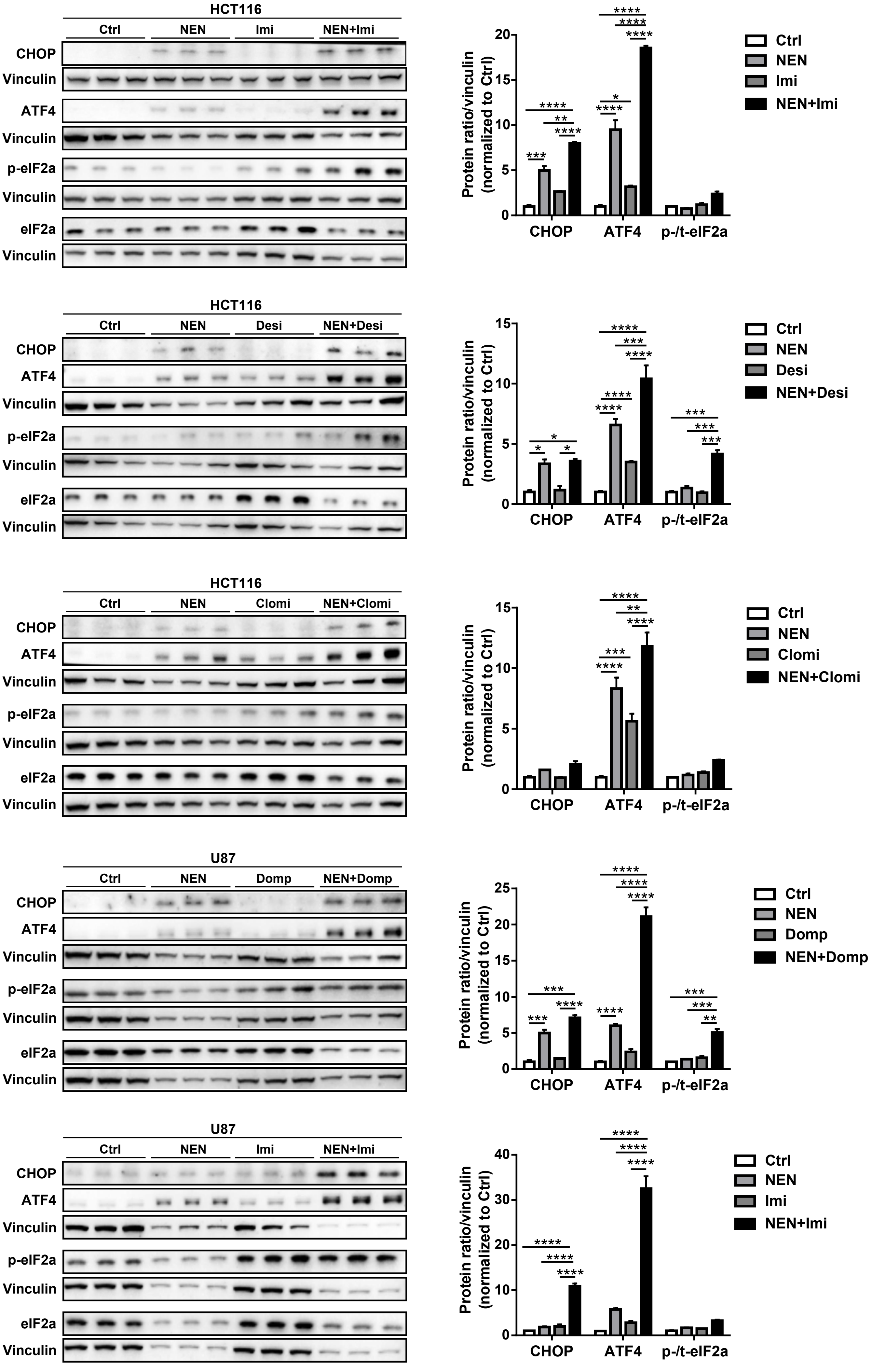

Appendix Figure S4B: Induction of ISR proteins upon drug treatments

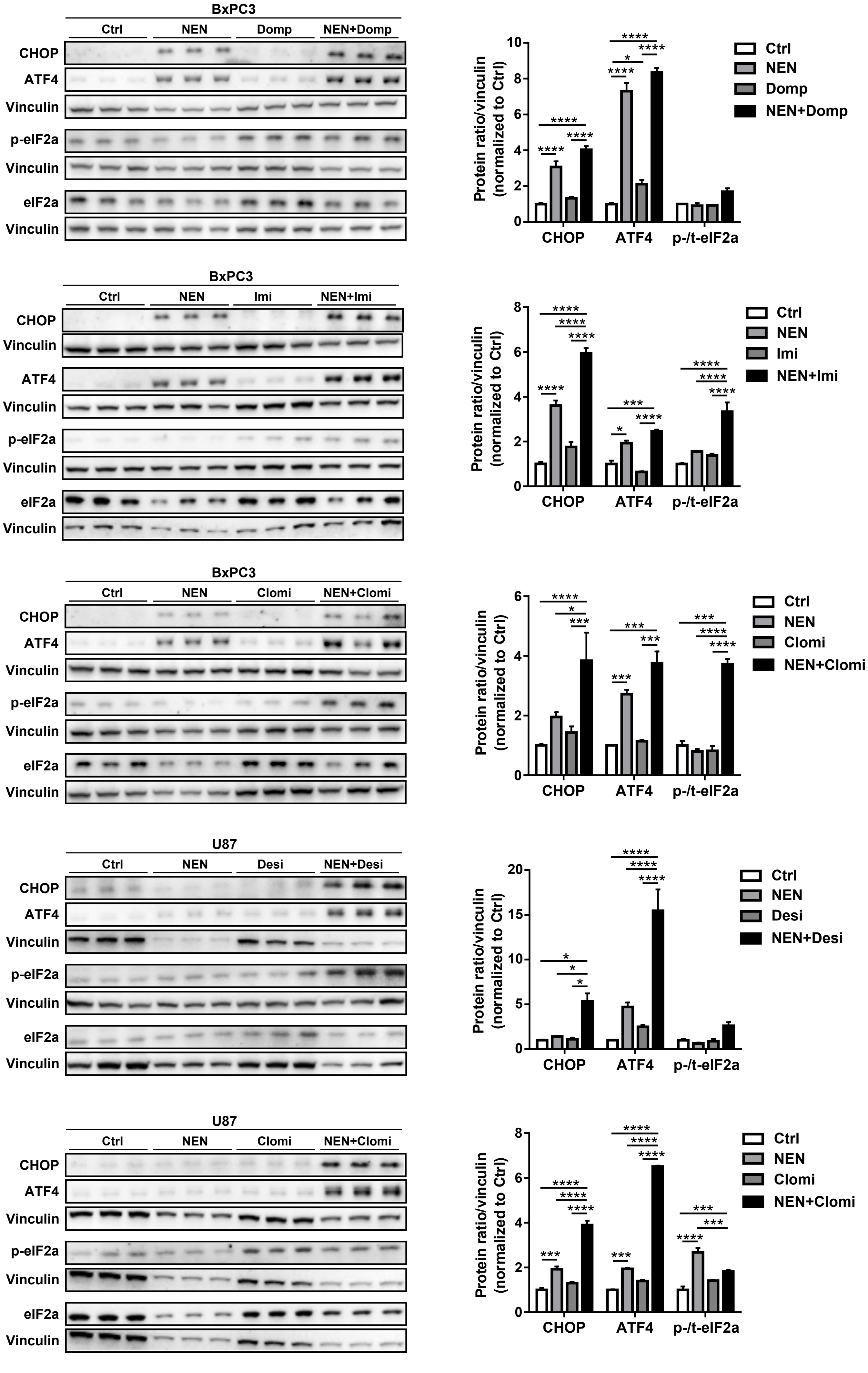

Appendix Figure S4A and for B

Legend:

A Immunoblot analysis including corresponding densitometric quantifications of CHOP, ATF4, phosphorylated (p-) and t-) eIF2alpha (eIF2a) of HCT116 and U87 cells treated as indicated for 16h or 24h, respectively (NEN 1.2 µM; Domperidone (Domp), Imipramine (Imi), Desipramine (Desi), each 30 µM; Clomipramine (Clomi) 20 µM).

B Immunoblot analysis including corresponding densitometric quantifications of CHOP, ATF4, phosphorylated (p-) and t-) eIF2alpha (eIF2a) of BxPC3 and U87 cells treated as indicated for 24h (NEN 1.2 µM; Domperidone (Domp), Imipramine (Imi), Desipramine (Desi), each 30 µM; Clomipramine (Clomi) 20 µM).

Data information: Data are presented as mean (SD) (N=3) and were analyzed two-way ANOVA with Tukey post-hoc test. * $p< 0.05$; ** $p< 0.01$;*** $p< 0.001$; **** $p < 0.0001$.

Appendix Figure S5: WikiPathways gene set enrichment analysis (GSEA) of transcriptome data

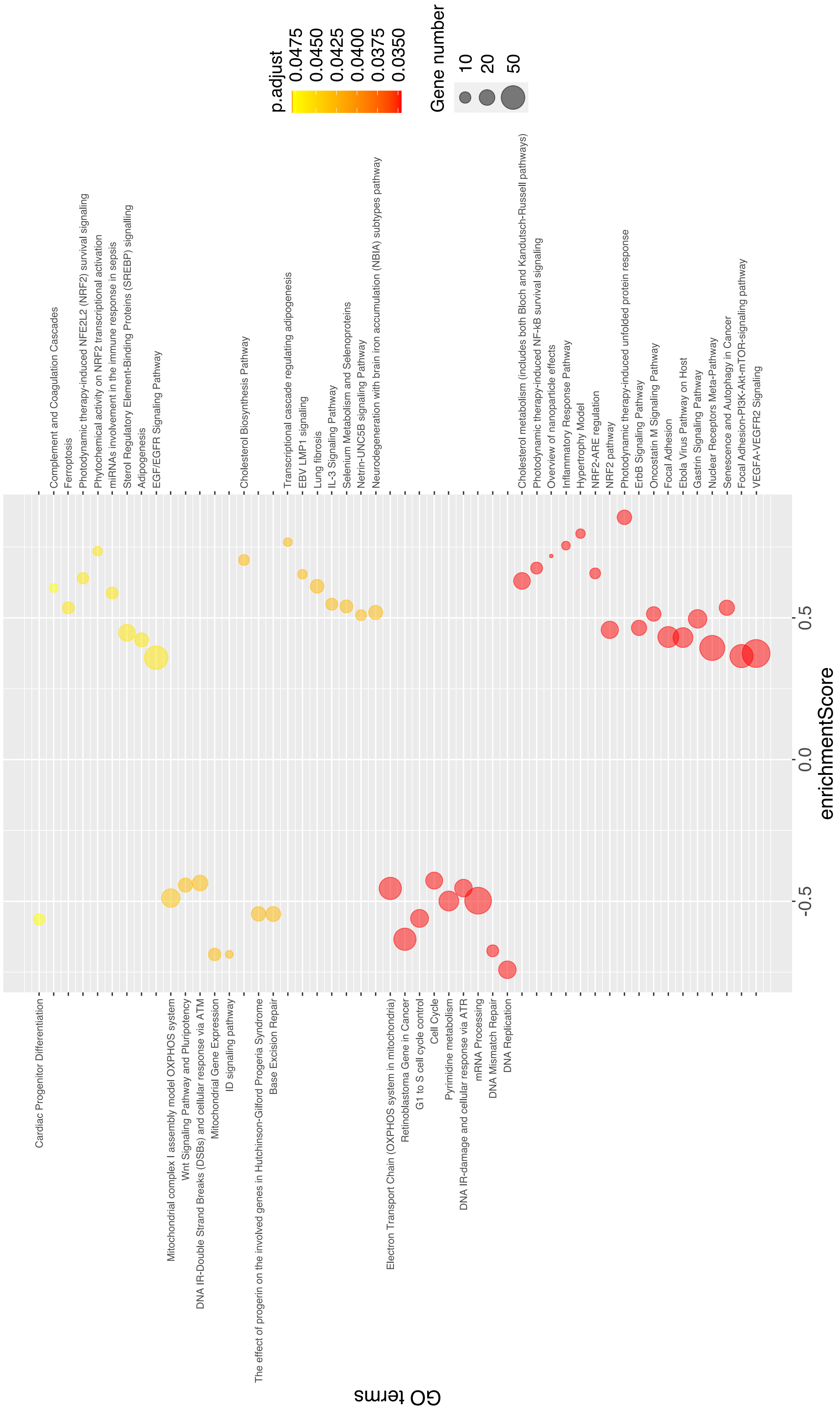

Legend: Wikipathway gene set enrichment analysis (GSEA) of microarray data from HCT116 cells upon double treatment with NEN (1.2µM) and Domperidone (30µM) for 16 hours vs. vehicle control treatment (N=3).

Appendix Figure S6: Heat map of CLEAR network gene expression from the transcriptome analysis

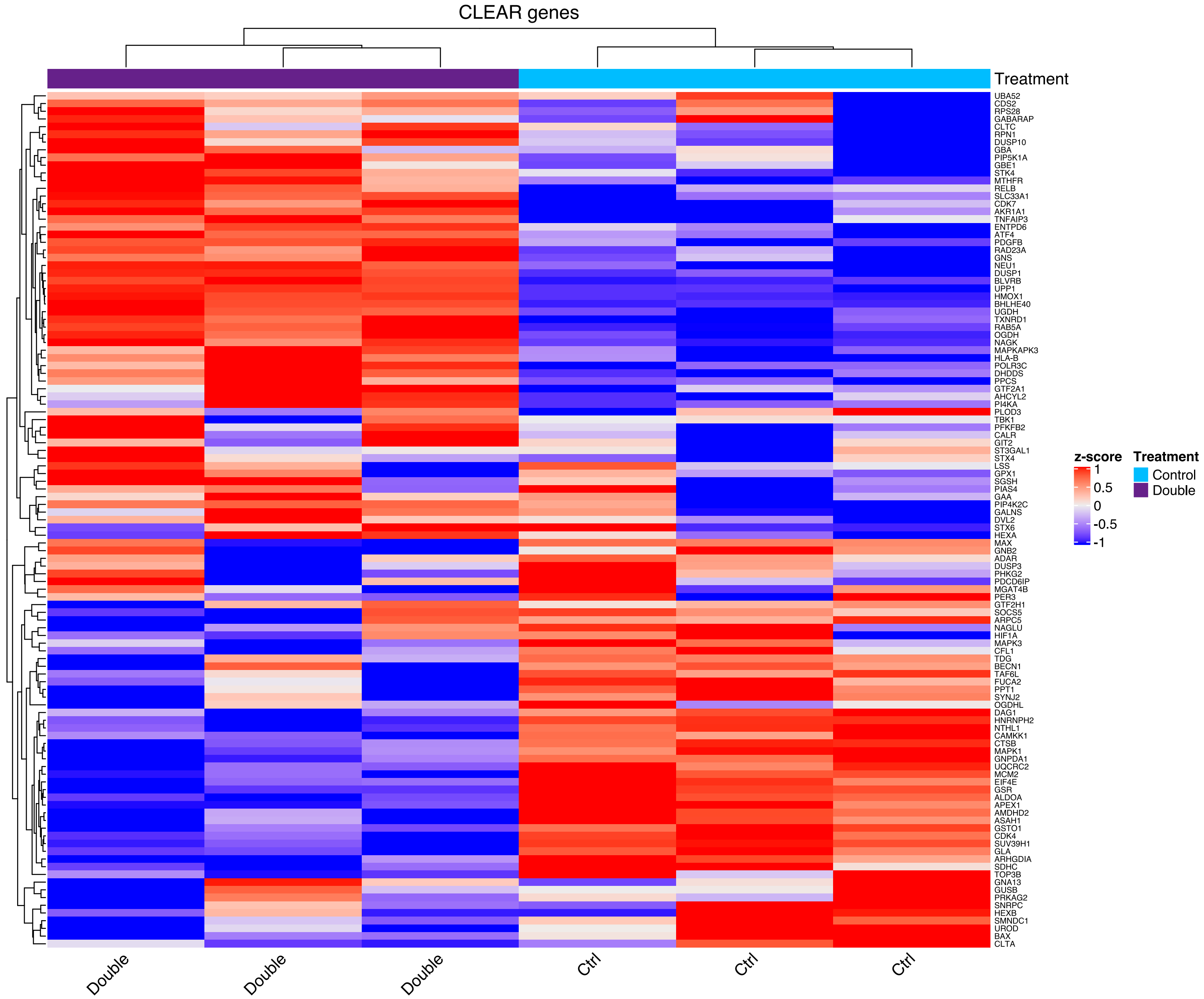

Legend: Expression of genes defining the CLEAR network as reported in Palmieri et al. (2011, Hum Mol Genet 20: 3852-3866, Table 2) from the microarray analysis of HCT116 cells upon treatment with vehicle control (Ctrl), NEN (1.2μM), Domperidone (Dom, 30μM) or double treatment with NEN and Domperidone for 16 hours. Of the 127 gene, 16 were missing from the expression matrix (probably filtered out due to low expression or they were missing from the Affymetrix chip).

Appendix Figure S7: Volcano blot of DEGs from the transcriptome analysis

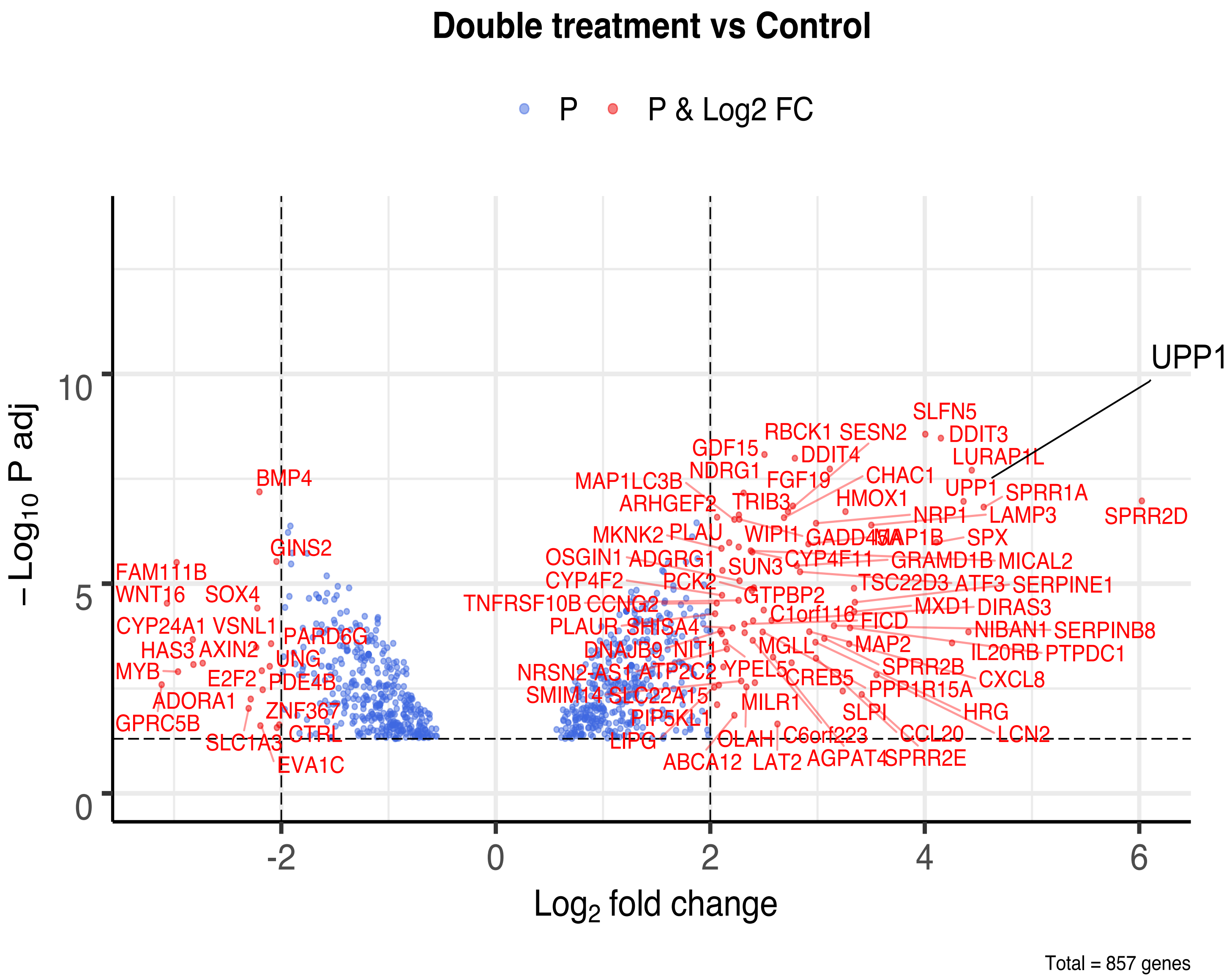

Legend: Volcano blot of differentially expressed genes determined from the microarray analysis from HCT116 cells upon double treatment with NEN (1.2µM) and Domperidone (30µM) for 16 hours vs. vehicle control treatment (N=3). UPP1 is highlighted as one of the strongest upregulated genes upon combined drug treatment.

Appendix Figure S8: Induction of UPP1 upon drug treatments

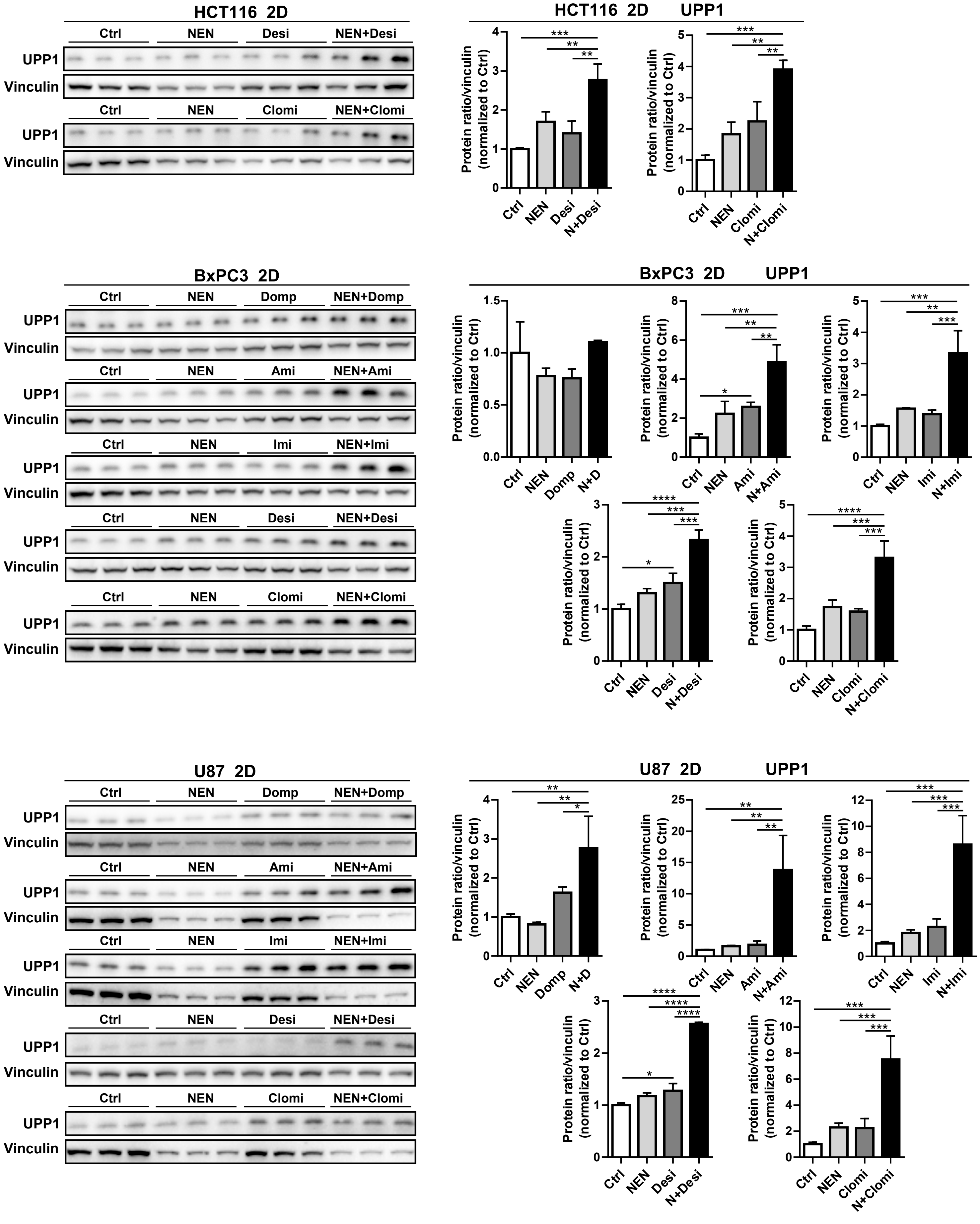

Legend:Immunoblot analysis including corresponding densitometric quantifications of UPP1 in HCT116, BxPC3 and U87 cells treated as indicated for 16h (HCT116) or 24h (BxPC3, U87) (NEN 1.2 µM Domepridone (Domp), Imipramine (Imi), Amitiptylin (Ami), 30µM each, and Clomipramine 20µM). Protein bands were normalized to vinculin (loading control) and were analyzed by one-way ANOVA and Tukey post-hoc test. * $p<0.05$; ** $p<0.01$; *** $p<0.001$; **** $p<0.0001$.

APPENDIX TABLE S1

Statistical tests and exact p-values

Figure panel	Test and post hoc test	p-value (main test)	Groups for post hoc comparisons	Adjusted p-values for multiple comparisons
Figure 1A	two-way ANOVA with Tukey post-hoc test	< 0.0001	NEN:Ctrl vs. NEN:2-DG<br>FCCP:Ctrl vs. FCCP:2-DG	0.0001<br>0.0337
Figure 1D	<u>HCT116:</u><br>two-way ANOVA with Tukey post-hoc test	< 0.0001	Ctrl:Domp vs. NEN:Domp<br>Ctrl:Imi vs. NEN:Imi<br>Ctrl:Ami vs. NEN:Ami<br>Ctrl:Desi vs. NEN:Desi<br>Ctrl:Clomi vs. NEN:Clomi	< 0.0001<br>< 0.0001<br>< 0.0001<br>< 0.0001<br>< 0.0001
	<u>U87:</u><br>two-way ANOVA with Tukey post-hoc test	< 0.0001	Ctrl:Ctrl vs. NEN:Ctrl<br>Ctrl:Domp vs. NEN:Domp<br>Ctrl:Imi vs. NEN:Imi<br>Ctrl:Ami vs. NEN:Ami<br>Ctrl:Desi vs. NEN:Desi<br>Ctrl:Clomi vs. NEN:Clomi	0.0332<br>< 0.0001<br>< 0.0001<br>0.0001<br>< 0.0001<br>< 0.0001
	<u>BxPC3:</u><br>two-way ANOVA with Tukey post-hoc test	< 0.0001	Ctrl:Domp vs. NEN:Domp<br>Ctrl:Imi vs. NEN:Imi<br>Ctrl:Ami vs. NEN:Ami<br>Ctrl:Desi vs. NEN:Desi<br>Ctrl:Clomi vs. NEN:Clomi	< 0.0001<br>< 0.0001<br>< 0.0001<br>0.4315 (ns)<br>< 0.0001
Figure 1E	<u>HCT116:</u><br>one-way ANOVA with Tukey post-hoc test	< 0.0001	Ctrl vs. NEN<br>Ctrl vs. N + D<br>Ctrl vs. N + D + zVAD<br>Ctrl vs. Staurospor.<br>N + D vs. N + D + zVAD<br>N + D vs. Staurospor.	0.0002<br>< 0.0001<br>< 0.0001<br>< 0.0001<br>0.0007<br>0.0052
	<u>U87:</u><br>Kruskal-Wallis and Dunn´s post-hoc test	0.0075	Ctrl vs. N + D<br>Domp vs. N + D	0.0206<br>0.0050

Figure 2A	HCT116; CHOP:<br>(upper left graph)<br>one-way ANOVA with Tukey post-hoc test<br>(upper right graph)<br>Kruskal-Wallis and Dunn´s post-hoc test<br><br>HCT116; ATF4:<br>(lower left graph)<br>one-way ANOVA with Tukey post-hoc test<br>(lower right graph)<br>Kruskal-Wallis and Dunn´s post-hoc test	< 0.0001<br><br>< 0.0001<br><br>< 0.0001<br><br>< 0.0001	Ctrl vs. N+D<br>NEN vs. N+D<br>Domp vs. N+D<br><br>Ctrl vs. NEN<br>Ctrl vs. NEN+Imi<br>Ctrl vs. NEN+Ami<br>Ctrl vs. NEN+Desi<br>Ctrl vs. NEN+Clomi<br><br>Ctrl vs. N+D<br>NEN vs. N+D<br>Domp vs. N+D<br><br>Ctrl vs. NEN+Imi<br>Ctrl vs. NEN+Ami<br>Ctrl vs. NEN+Desi<br>Ctrl vs. NEN+Clomi	< 0.0001<br>< 0.0001<br>< 0.0001<br><br>0.0355<br>0.0049<br>0.0003<br>0.0001<br>0.0004<br><br>< 0.0001<br>< 0.0001<br>0.0004<br><br>0.0088<br>0.0017<br>0.0003<br>0.0011
Figure 2C	two-way ANOVA with Tukey post-hoc test	< 0.0001	Ctrl:Crtl-siRNA vs. N+D:Crtl-siRNA<br>N+D:Crtl-siRNA vs. N+D:siATF4<br>N+D:Crtl-siRNA vs. N+D:siCHOP	< 0.0001<br>0.1369 (ns)<br>< 0.0001
Figure 2D	one-way ANOVA with Tukey post-hoc test	GADD34<br><0.0001<br><br>CHOP<br><br><br>ATF4	GADD34<br>Ctrl vs. N+D<br>N+D vs. N+D+ISRIB<br>CHOP<br>Ctrl vs. N+D<br>N+D vs. N+D+ISRIB<br>ATF4<br>Ctrl vs. N+D<br>N+D vs. N+D+ISRIB	<0.0001<br>0.0001<br><br>< 0.0001<br>< 0.0001<br><br>0.0002<br>0.0046
Figure 2E	two-way ANOVA with Tukey post-hoc test	< 0.0001	Ctrl:Ctrl vs. NEN:Domperidone<br>NEN:Domperidone vs. NEN:ISRIB	< 0.0001<br>< 0.0001
Figure 2F	two-way ANOVA with Tukey post-hoc test	<0.0001	Ctrl:NEN+Domp vs. ISRIB:NEN+Domp<br>Ctrl:NEN+Imi vs. ISRIB:NEN+Imi<br>Ctrl:NEN+Ami vs. ISRIB:NEN+Ami<br>Ctrl:NEN+Desi vs. ISRIB:NEN+Desi<br>Ctrl:NEN+Clomi vs. ISRIB:NEN+Clomi	< 0.0001<br>< 0.0001<br>< 0.0001<br>< 0.0001<br>< 0.0001

Figure 3A	TFE3: (left graph):			
	one-way ANOVA	<0.0001	Ctrl vs. NEN	< 0.0001
	with Tukey post-		Ctrl vs. Domp	0.0118
	hoc test		Ctrl vs. N+D	<0.0001
			NEN vs. N+D	<0.0001
			Domp vs. N+D	<0.0001
	TFE3 (right graph):			
	one-way ANOVA	<0.0001	Ctrl vs. NEN+Imi	0.0052
	with Tukey post-		Ctrl vs. NEN+Ami	0.0482
	hoc test		Ctrl vs. NEN+Desi	<0.0001
			Ctrl vs. NEN+Clomi	<0.0001
	CD68 (left graph):			
	one-way ANOVA	<0.0001	Ctrl vs. NEN	0.0002
	with Tukey post-		Ctrl vs. Domp	<0.0001
	hoc test		Ctrl vs. N+D	<0.0001
	CD68 (right graph):			
	one-way ANOVA	<0.0001	Ctrl vs. NEN+Imi	<0.0001
	with Tukey post-		Ctrl vs. NEN+Ami	<0.0001
	hoc test		Ctrl vs. NEN+Desi	<0.0001
			Ctrl vs. NEN+Clomi	<0.0001
Figure 3B	Lamp1 (top panel):			
	Kruskal-Wallis with	<0.0001	Ctrl vs. NEN	0.0008
	Dunn´s post-hoc		Ctrl vs. NEN+Domp	<0.0001
	test		NEN vs. NEN+Domp	0.0057
			Domp vs. NEN+Domp	<0.0001
	LC3 (middle panel):			
	one-way ANOVA	<0.0001	Ctrl vs. NEN	<0.0001
	with Tukey post-		Ctrl vs. NEN+Domp	<0.0001
	hoc test		NEN vs. Domp	0.0468
			NEN vs. NEN+Domp	<0.0001
			Domp vs. NEN+Domp	<0.0001
	p62 (lower panel):			
	Kruskal-Wallis with	<0.0001	Ctrl vs. NEN	0.0008
	Dunn´s post-hoc		NEN vs. Domp	<0.0001
	test		Domp vs. NEN+Domp	<0.0001
Figure 3C	LC3-II/I protein			
	ratio:			
	one-way ANOVA	<0.0001	Ctrl vs. NEN	0.0387
	with Tukey post-		Ctrl vs. NEN+Domp	<0.0001
	hoc test		NEN vs. NEN+Domp	<0.0001
	p62 levels:			
	one-way ANOVA	<0.0001	Ctrl vs. NEN+Domp	<0.0001
	with Tukey post-		NEN vs. NEN+Domp	0.0002
	hoc test			
Figure 3D	Kruskal-Wallis with	<0.0001	Ctrl vs. NEN	0.0461
	Dunn´s post-hoc		Ctrl vs. NEN+Domp	0.0007
	test			
Figure 4B	UPP1, left graph:			
	one-way ANOVA	<0.0001	Ctrl vs. Domp	0.0002
	with Tukey post-		Ctrl vs. N+D	0.0002
	hoc test		NEN vs. N+D	<0.0001

	<u>UPP1, right graph:</u><br>one-way ANOVA with Tukey post-hoc test	<0.0001	Domp vs. N+D<br><br>Ctrl vs. NEN+Imi<br>Ctrl vs. NEN+Ami<br>Ctrl vs. NEN+Desi<br>Ctrl vs. NEN+Clomi	<0.0001<br><br><0.0001<br><0.0001<br><0.0001<br><0.0001
Figure 4C	two-way ANOVA with Tukey post-hoc test	0.0016 (treatment)<br>0.0137 (KD)	Ctrl:siCtrl vs. N+D:siCtrl<br>N+D:siCtrl vs. N+D+ISRIB:siCtrl<br>N+D:siCtrl vs. N+D:siTFE3/MITF	0.0024<br>0.0041<br>0.0071
Figure 4D	two-way ANOVA with Tukey post-hoc test	<0.0001	siCtrl:Ctrl vs. siCtrl:N+D<br>siCtrl:N+D vs. siUPP1:N+D	<0.0001<br><0.0001
Figure 4E	3D Toxicity (left graph):<br>two-way ANOVA with Tukey post-hoc test<br><br>2D Toxicity (right graph):<br>two-way ANOVA with Tukey post-hoc test	<0.0001<br><br><0.0001	siCtrl:Ctrl vs. siCtrl:NEN+Domp<br>siCtrl:NEN+Domp vs. siUPP1:NEN+Domp<br>siCtrl:Ctrl vs. siUPP1:NEN+Domp<br>siCtrl:NEN+Domp vs. siUPP1:NEN+Domp	<0.0001<br>0.0001<br><0.0001<br><0.0001
Figure 4H	two-way ANOVA with Tukey post-hoc test		siCtrl:NEN+Imi vs. siUPP1:NEN+Imi<br>siCtrl:NEN+Ami vs. siUPP1:NEN+Ami<br>siCtrl:NEN+Desi vs. siUPP1:NEN+Desi	0.0003<br><0.0001<br>0.0004
Figure 5A	<u>DHODH, Left graph:</u><br>Kruskal-Wallis with Dunn´s post-hoc test<br><br><u>DHODH, right graph:</u><br>one-way ANOVA with Tukey post-hoc test	<0.0001	Ctrl vs. NEN<br>Ctrl vs. N+D<br><br>Ctrl vs. NEN<br>Ctrl vs. Imi<br>Ctrl vs. NEN+Imi<br>Ctrl vs. Ami<br>Ctrl vs. NEN+Ami<br>Ctrl vs. NEN+Desi<br>Ctrl vs. Clomi<br>Ctrl vs. NEN+Clomi	0.0067<br>0.1246<br><br><0.0001<br><0.0001<br><0.0001<br><0.0001<br><0.0001<br><0.0001<br>0.0061<br><0.0001
Figure 5B	two-way ANOVA with Tukey post-hoc test	<0.0001	Ctrl+Jnki:Ctrl vs. NEN:Domp+A77	<0.0001

			NEN:Domp vs.<br>NEN:Domp+A77<br>NEN:Domp+A77 vs.<br>NEN+Jnki:Domp+A77	<0.0001<br><br><0.0001
Figure 5D	Left graph:<br>two-way ANOVA with Tukey post-hoc test<br><br>Right graph:<br>two-way ANOVA with Tukey post-hoc test	<0.0001<br><br><0.0001	NEN:Imipramine vs.<br>NEN:Imi+A77<br>NEN:Desipramine vs.<br>NEN:Desi+A77<br>NEN:Amitriptyline vs.<br>NEN:Ami+A77<br><br>Ctrl:Clomi+A77 vs.<br>NEN:Clomi+A77<br>NEN:Clomi vs.<br>NEN:Clomi+A77	<0.0001<br><br><0.0001<br><br><0.0001<br><br><0.0001<br><br><0.0001
Figure 6C	two-way ANOVA with Tukey post-hoc test	0.0002	Ctrl:Ctrl vs. Ctrl:NEN+Domp<br>Ctrl:NEN+Domp vs.<br>0.3%CD:NEN+Domp<br>Ctrl:NEN+Domp vs.<br>1.5%CD:NEN+Domp<br>0.01%CD:Ctrl vs.<br>0.01%CD:NEN+Domp<br>0.06%CD:Ctrl vs.<br>0.06%CD:NEN+Domp	<0.0001<br><br>0.0004<br><br><0.0001<br><br>0,0007<br><br>0,0004
Figure 6D	two-way ANOVA with Tukey post-hoc test	<0.0001	Ctrl:NEN+Domp vs.<br>CD:NEN+Domp<br>Ctrl:NEN+Imi vs.<br>CD:NEN+Imi<br>Ctrl:NEN+Ami vs.<br>CD:NEN+Ami<br>Ctrl:NEN+Desi vs.<br>CD:NEN+Desi<br>Ctrl:NEN+Clomi vs.<br>CD:NEN+Clomi	<0.0001<br><br>0.0009<br><br><0.0001<br><br><0.0001<br><br><0.0001
Figure 6E	CHOP:<br>one-way ANOVA with Tukey post-hoc test<br><br>TFE3:<br>one-way ANOVA with Tukey post-hoc test<br><br>CD68:<br>one-way ANOVA with Tukey post-hoc test<br><br>NPC1:	<0.0001<br><br>0.0030<br><br><0.0001	Ctrl vs. N+D<br>N+D vs. N+D+CyD<br><br>Ctrl vs. N+D<br><br>Ctrl vs. N+D<br>N+D vs. N+D+CyD	<0.0001<br><0.0001<br><br>0.0014<br><br><0.0001<br>0.0323

	one-way ANOVA with Tukey post-hoc test<br><br>UPP1:<br>one-way ANOVA with Tukey post-hoc test<br><br>DHODH:<br>one-way ANOVA with Tukey post-hoc test	0.0001<br><br><br><br><br><0.0001<br><br><br><br><br>0.0038	Ctrl vs. N+D<br>N+D vs. N+D+CyD<br><br><br><br>Ctrl vs. N+D<br>N+D vs. N+D+CyD<br><br><br><br>Ctrl vs. N+D	<0.0001<br>0.0078<br><br><br><br><0.0001<br><0.0001<br><br><br><br>0.0082
Figure 7A	PDO-42 (left):<br>one-way ANOVA with Tukey post-hoc test<br><br><br><br>PDO-48 (right):<br>one-way ANOVA with Tukey post-hoc test	<0.0001<br><br><br><br><br><br>0.0003	Ctrl vs. NEN 1,2+A<br>Imi30 vs. NEN1,2 + I<br>Desi30 vs. NEN1,2+D<br>NEN2,5 vs. NEN2,5+C20<br><br><br>Ami vs. NEN 1,2+A<br>Desi vs. NEN1,2+D<br>NEN 1,2 vs. NEN1,2+C<br>NEN 2,5 vs. NEN 2,5+A<br>NEN 2,5 vs. NEN 2,5+I<br>NEN 2,5 vs. NEN 2,5+D<br>NEN 2,5 vs. NEN 2,5+C	0.0001<br>0.0001<br><0.0001<br>0.0041<br><br><br>0.0331<br>0.0456<br>0.0257<br>0.0016<br>0.0003<br>0.0001<br><0.0001
Figure 7B	PDO-42:<br>one-way ANOVA with Tukey post-hoc test<br><br><br><br><br><br><br><br><br><br><br><br>PDO-48:<br>one-way ANOVA with Tukey post-hoc test	<0.0001<br><br><br><br><br><br><br><br><br><br><br><br><br><0.0001	Upper left graph:<br>Ctrl vs. N+A<br>Ctrl vs. N+A+Paclitaxel 2nM<br>Ctrl vs. N+Paclitaxel 20nM<br>Ctrl vs. N+A+Paclitaxel 20nM<br>Lower left graph:<br>Ctrl vs. N+I+Paclitaxel 2nM<br>Ctrl vs. N+Paclitaxel 20nM<br>Ctrl vs. N+I+ Paclitaxel 20nM<br>Ctrl vs. Pacli 200<br><br>Upper right graph<br>Ctrl vs. N+A+Paclitaxel 2nM<br>Ctrl vs. Pacli 20<br>Ctrl vs. N+Paclitaxel 20nM<br>Ctrl vs. N+A+Paclitaxel 20nM<br>Ctrl vs. Pacli 200<br>Lower left graph:<br>Ctrl vs. Pacli 20	 0.0372<br><0.0001<br>0.0016<br><0.0001<br>0.0010<br><br>0.0019<br>0.0005<br><br><0.0001<br>0.0003<br><br><br>0.0154<br>0.0006<br><0.0001<br><br><0.0001<br><0.0001<br><br>0.0008

			Ctrl vs. N+Paclitaxel 20nM<br>Ctrl vs. N+I+ Paclitaxel 20nM<br>Ctrl vs. Pacli 200	<0.0001<br><br><0.0001<br><0.0001
Figure EV1A	BxPC3 (upper graph):<br>two-way ANOVA with Tukey post-hoc test<br>U87 (lower graph):<br>two-way ANOVA with Tukey post-hoc test	< 0.0001<br><br>< 0.0001	NEN:Ctrl vs. NEN:2-DG<br>NEN:Ctrl vs. NEN:2-DG<br>FCCP:Ctrl vs. FCCP:2-DG<br><br>NEN:Ctrl vs. NEN:2-DG<br>NEN:Ctrl vs. NEN:2-DG<br>FCCP:Ctrl vs. FCCP:2-DG	<0.0001<br><0.0001<br><0.0001<br><br><0.0001<br><0.0001<br><0.0001
Figure EV1B	one-way ANOVA with Tukey post-hoc test	< 0.0001	Ctrl vs. 2-DG<br>Ctrl vs. NEN+2-DG<br>NEN vs. 2-DG<br>NEN vs. NEN+2-DG<br>2-DG vs. NEN+2-DG	<0.0001<br><0.0001<br><0.0001<br><0.0001<br><0.0001
Figure EV1C	one-way ANOVA with Tukey post-hoc test	< 0.0001	Ctrl 1% vs. NEN 10%<br>NEN 10% vs. NEN 1%	<0.0001<br><0.0001
Figure EV1D	HCT116, upper graph:<br>two-way ANOVA with Tukey post-hoc test<br><br>BxPC3, lower graph:<br>two-way ANOVA with Tukey post-hoc test	< 0.0001<br><br>< 0.0001	Ctrl:Domp vs. NEN:Domp<br>Ctrl:Imi vs. NEN:Imi<br>Ctrl:Ami vs. NEN:Ami<br>Ctrl:Desi vs. NEN:Desi<br>Ctrl:Clomi vs. NEN:Clomi<br><br>Ctrl:Imi vs. NEN:Imi<br>Ctrl:Ami vs. NEN:Ami<br>Ctrl:Desi vs. NEN:Desi<br>Ctrl:Clomi vs. NEN:Clomi	<0.0001<br><0.0001<br><0.0001<br><0.0001<br><0.0001<br><br><0.0001<br><0.0001<br><0.0001<br><0.0001
Figure EV1E	HCT116, upper graph:<br>two-way ANOVA with Tukey post-hoc test<br><br>U87, lower graph:<br>two-way ANOVA with Tukey post-hoc test	< 0.0001<br><br>< 0.0001	Ctrl:Ctrl vs. NEN:Ctrl<br>Ctrl:Domp vs. NEN:Domp<br>Ctrl:Imi vs. NEN:Imi<br>Ctrl:Ami vs. NEN:Ami<br>Ctrl:Desi vs. NEN:Desi<br>Ctrl:Clomi vs. NEN:Clomi<br><br>Ctrl:Domp vs. NEN:Domp<br>Ctrl:Imi vs. NEN:Imi<br>Ctrl:Ami vs. NEN:Ami<br>Ctrl:Desi vs. NEN:Desi	<0.0001<br><0.0001<br><0.0001<br><0.0001<br><0.0001<br><0.0001<br><br><0.0001<br><0.0001<br><0.0001<br><0.0001

			Ctrl:Clomi vs. NEN:Clomi	<0.0001
Figure EV1F	one-way ANOVA+Welch´s correction with Dunnet´s post hoc test	< 0.0001	Ctrl vs. Staurosp	<0.0001
Figure EV2A	one-way ANOVA with Dunnet´s post hoc test		Ctrl vs. NEN+Imi<br>Ctrl vs. NEN+Ami<br>Ctrl vs. NEN+Desi<br>Ctrl vs. NEN+Clomi	0.0096<br>0.0002<br><0.0001<br><0.0001
Figure EV2C	<u>HCT116, left graph:</u><br>two-way ANOVA with Tukey post-hoc test	<0.0001	CHOP:Ctrl vs. CHOP:NEN<br>CHOP:Ctrl vs. CHOP:NEN+Domp<br>ATF4:Ctrl vs. ATF4:NEN+Domp<br>p-/t-eIF2a:Ctrl vs. p-/t-eIF2a:NEN+Domp<br>p-/t-eIF2a:NEN vs. p-/t-eIF2a:NEN+Domp	0.0003<br><0.0001<br>0.0007<br>0.0006<br>0.0005
	<u>HCT116, middle graph:</u><br>two-way ANOVA with Tukey post-hoc test	<0.0001	CHOP:Ctrl vs. CHOP:NEN<br>CHOP:Ctrl vs. CHOP:NEN+Ami<br>CHOP:NEN vs. CHOP:NEN+Ami<br>CHOP:Ami vs. CHOP:NEN+Ami<br>ATF4:Ctrl vs. ATF4:NEN+Ami<br>ATF4:NEN vs. ATF4:NEN+Ami<br>ATF4:Ami vs. ATF4:NEN+Ami<br>p-/t-eIF2a:Ctrl vs. p-/t-eIF2a:NEN+Ami<br>p-/t-eIF2a:NEN vs. p-/t-eIF2a:NEN+Ami<br>p-/t-eIF2a:Ami vs. p-/t-eIF2a:NEN+Ami	0.0010<br><0.0001<br>0.0062<br><0.0001<br><0.0001<br><0.0001<br>0.0002<br><0.0001<br><0.0001<br><0.0001
	<u>U87, right graph:</u><br>two-way ANOVA with Tukey post-hoc test	<0.0001	CHOP:Ctrl vs. CHOP:NEN+Ami<br>CHOP:NEN vs. CHOP:NEN+Ami<br>CHOP:Ami vs. CHOP:NEN+Ami<br>ATF4:Ctrl vs. ATF4:NEN<br>ATF4:Ctrl vs. ATF4:NEN+Ami	<0.0001<br><0.0001<br><0.0001<br><0.0001<br><0.0001

			ATF4:NEN vs. ATF4:NEN+Ami<br>ATF4:Ami vs. ATF4:NEN+Ami<br>p-/t-eIF2a:Ctrl vs. p-/t-eIF2a:NEN+Ami<br>p-/t-eIF2a:NEN vs. p-/t-eIF2a:NEN+Ami<br>p-/t-eIF2a:Ami vs. p-/t-eIF2a:NEN+Ami	<0.0001<br><0.0001<br><0.0001<br>0.0266<br>0.0012
Figure EV2D	Unpaired t test with Welch's correction		Left graph:<br>Ctrl vs. siATF4<br>Right graph:<br>Ctrl vs. siChop	 0.0015<br><br>0.0064
Figure EV2E	two-way ANOVA with Tukey post-hoc test	<0.0001	Ctrl:NEN+Imi vs. ISRIB:NEN+Imi<br>Ctrl:NEN+Ami vs. ISRIB:NEN+Ami<br>Ctrl:NEN+Desi vs. ISRIB:NEN+Desi<br>Ctrl:NEN+Clomi vs. ISRIB:NEN+Clomi	<0.0001<br><0.0001<br><0.0001<br>0.0020
Figure EV2F	two-way ANOVA with Tukey post-hoc test	<0.0001	Ctrl:NEN+Domp vs. ISRIB:NEN+Domp<br>Ctrl:NEN+Imi vs. ISRIB:NEN+Imi<br>Ctrl:NEN+Ami vs. ISRIB:NEN+Ami<br>Ctrl:NEN+Desi vs. ISRIB:NEN+Desi<br>Ctrl:NEN+Clomi vs. ISRIB:NEN+Clomi	<0.0001<br><0.0001<br><0.0001<br><0.0001<br>0.0017
Figure EV3A	MITF, left graph: one-way ANOVA with Tukey post-hoc test<br>MITF, right graph: one-way ANOVA with Tukey post-hoc test<br>SQSTM1, left graph: one-way ANOVA with Tukey post-hoc test<br>SQSTM1, right graph: one-way	0.0006<br>0.0001<br><0.0001<br><0.0001	Ctrl vs. NEN<br>Ctrl vs. Domp<br>Ctrl vs. N+D<br>Ctrl vs. NEN+Clomi<br>Ctrl vs. NEN<br>Ctrl vs. Domp<br>Ctrl vs. N+D<br>NEN vs. N+D<br>Domp vs. N+D<br>Ctrl vs. NEN+Imi<br>Ctrl vs. NEN+Ami	0.0009<br>0.0024<br>0.0012<br>0.0220<br>0.0031<br><0.0001<br><0.0001<br><0.0001<br>0.0004<br><0.0001<br>0.0007

	ANOVA with Tukey post-hoc test<br><br><u>NPC1, left graph:</u> one-way ANOVA with Tukey post-hoc test<br><u>NPC1, right graph:</u> one-way ANOVA with Tukey post-hoc test	 <br><br>0.0098<br><br><br><br><0.0001	Ctrl vs. NEN+Desi<br>Ctrl vs. NEN+Clomi<br><br>Ctrl vs. Domp<br>Ctrl vs. N+D<br><br><br>Ctrl vs. NEN+Imi<br>Ctrl vs. NEN+Ami<br>Ctrl vs. NEN+Desi<br>Ctrl vs. NEN+Clomi	<0.0001<br><0.0001<br><br>0.0077<br>0.0405<br><br><br><0.0001<br><0.0001<br><0.0001<br><0.0001
Figure EV3C	<u>LC3-II/I protein ratio:</u><br>one-way ANOVA with Tukey post-hoc test<br><u>p62 levels:</u><br>one-way ANOVA with Tukey post-hoc test	 <br><0.0001<br><br><br><br><0.0001	 <br>Ctrl vs. NEN<br>Ctrl vs. NEN+IMI<br>NEN vs. NEN+IMI<br><br>Ctrl vs. NEN<br>Ctrl vs. NEN+IMI<br>NEN vs. NEN+IMI	 <br>0.0007<br><0.0001<br><0.0001<br><br>0.0402<br>0.0003<br>0.0030
Figure EV3D	<u>LC3-II/I protein ratio:</u><br>one-way ANOVA with Tukey post-hoc test<br><u>p62 levels:</u><br>one-way ANOVA with Tukey post-hoc test	 <br><0.0001<br><br><br><br><0.0001	 <br>Ctrl vs. NEN<br>Ctrl vs. NEN+AMI<br>NEN vs. NEN+AMI<br><br>Ctrl vs. NEN<br>Ctrl vs. NEN+AMI<br>NEN vs. NEN+AMI	 <br>0.0005<br><0.0001<br><0.0001<br><br>0.0551 (ns)<br>0.0006<br>0.0077
Figure EV4A	one-way ANOVA with Tukey post-hoc test	<0.0001	Ctrl vs. N+D<br>NEN vs. N+D<br>Domp vs. N+D	<0.0001<br><0.0001<br><0.0001
Figure EV4C	<u>UPP1, left graph:</u><br>one-way ANOVA with Tukey post-hoc test<br><br><br><u>UPP1, middle graph:</u><br>one-way ANOVA with Tukey post-hoc test<br><br><u>UPP1, right graph:</u><br>one-way ANOVA with Tukey post-hoc test	 <0.0001<br><br><br><br><br><br><br>0.0017<br><br><br><br><br>0.0002	 Ctrl vs. NEN<br>Ctrl vs. Domp<br>Ctrl vs. N+D<br>NEN vs. N+D<br>Domp vs. N+D<br><br><br>Ctrl vs. N+Ami<br>NEN vs. N+Ami<br>Ami vs. N+Ami<br><br><br>Ctrl vs. N+Imi<br>NEN vs. N+Imi<br>Ami vs. N+Imi	 0.0123<br>0.0006<br><0.0001<br>0.0003<br>0.0050<br><br><br>0.0017<br>0.0079<br>0.0053<br><br><br>0.0003<br>0.0010<br>0.0011

Figure EV4F	two-way ANOVA with Tukey post-hoc test	<0.0001	siCtrl:NEN+Domp vs. siUPP1:NEN+Domp<br>siCtrl:NEN+Imi vs. siUPP1:NEN+Imi<br>siCtrl:NEN+Ami vs. siUPP1:NEN+Ami<br>siCtrl:NEN+Desi vs. siUPP1:NEN+Desi<br>siCtrl:NEN+Clomi vs. siUPP1:NEN+Clomi	0.0034<br>0.0211<br>0.0135<br>0.0196<br>0.1278 (ns)
Figure EV5A	PDO-B42:<br>one-way ANOVA with Tukey post-hoc test<br><br>PDO-48:<br>one-way ANOVA with Tukey post-hoc test	<0.0001<br><br><br><0.0001	Upper left graph:<br>Ctrl vs. N+D+Paclitaxel 2nM<br>Ctrl vs. N+Paclitaxel 20nM<br>Ctrl vs. N+D+ Paclitaxel 20nM<br>Ctrl vs. Pacli 200<br>Lower left graph:<br>Ctrl vs. N+C+Paclitaxel 2nM<br>Ctrl vs. N+C+Paclitaxel 20nM<br><br>Upper right graph:<br>Ctrl vs. Pacli 20<br>Ctrl vs. N+Paclitaxel 20nM<br>Ctrl vs. N+D+Paclitaxel 20nM<br>Ctrl vs. Pacli 200<br>Lower right graph:<br>Ctrl vs. N+C+Paclitaxel 2nM<br>Ctrl vs. Pacli 20<br>Ctrl vs. N+Paclitaxel 20nM<br>Ctrl vs. N+C+Paclitaxel 20nM<br>Ctrl vs. Pacli 200	 0.0053<br>0.0275<br><0.0001<br>0.0203<br><br>0.0113<br><0.0001<br><br><br>0.0068<br><0.0001<br><0.0001<br><0.0001<br><br>0.0010<br>0.0024<br><0.0001<br><0.0001<br><0.0001
Figure EV5B	Left graph, CHOP:<br>one-way ANOVA with Dunnet´s post hoc test<br>Middle graph, UPP1:<br>one-way ANOVA with Dunnet´s post hoc test<br>Right graph, PUMA:<br>one-way ANOVA with Dunnet´s post hoc test	0,0148<br><br>0.0061<br><br>0,0039	Ctrl vs. NEN+Ami<br>NEN vs. NEN+Ami<br>Ami vs. NEN+Ami<br><br>Ctrl vs. NEN+Ami<br>NEN vs. NEN+Ami<br>Ami vs. NEN+Ami<br><br>Ctrl vs. NEN+Ami<br>NEN vs. NEN+Ami<br>Ami vs. NEN+Ami	0,0316<br>0,0578 (ns)<br>0,0345<br><br>0.0307<br>0.1257<br>0.0184<br><br>0,0058<br>0,1306<br>0,1430
