# Supplementary material for: Combination therapies induce cancer cell death through the integrated stress response and disturbed pyrimidine metabolism
Source: EMBO Mol Med. 2021 Mar 5;13(4):e12461. doi: 10.15252/emmm.202012461 (PMC8033521; doi:10.15252/emmm.202012461)
Supplement: Supplementary file 2 — Expanded View Figures PDF [file EMMM-13-e12461-s005.pdf]

# Expanded View Figures

**Figure EV1. Mitochondrial uncoupling induces secondary vulnerabilities.**

A BxPC3 and U87 cells grown in monolayer were treated as indicated (Control (Ctrl), 2-DG 100 mM; NEN 1.2 μM; FCCP 2.5 μM). Graph shows the ratio of toxicity and viability measured after an 18 h treatment.
B HCT116 cells were treated as indicated (NEN 1.2 μM; 2-DG 100 mM) for 8 h and ATP content was measured.
C Caspase activity of HCT116 cells grown in 10 or 1% serum supplemented medium and treated as indicated for 18 h treatment (NEN 1. 2 μM).
D Caspase activity in HCT116 and BxPC3 cells grown as monolayer (2D) treated as indicated for 18 or 48 h, respectively (NEN 1.2 μM; domperidone (Domp), imipramine (Imi), desipramine (Desi), and amitriptyline (Ami), each 30 μM; clomipramine (Clomi) 20 μM).
E Percentage of apoptotic cells, determined by measuring Annexin V- and PI-positive HCT116 or U87 cells by FACS. The effects were assessed after a 24-h or 48-h treatment, respectively (NEN 1.2 μM; domperidone (Domp), imipramine (Imi), desipramine (Desi), and amitriptyline (Ami), each 30 μM; clomipramine (Clomi) 20 μM).
F Caspase activity of primary hepatocytes treated as indicated for 24 h (NEN (N) 1.2 μM; domperidone (Domp), imipramine (Imi), desipramine (Desi), and amitriptyline (Ami), each 30 μM; clomipramine (Clomi) 20 μM). Staurosporine (100 μM) was used as positive control.

Data information: In (A ($N$ = 3), B ($N$ = 5), C ($N$ = 3), D ($N$ = 3), E ($N$ = 3), F (for Ctrl, NEN, Domp, NEN + Domp ($N$ = 6); for Imi, Ami, Desi, Clomi, N + Imi, N + Ami, N + Desi, N + Clomi ($N$ = 3); for Saurospo ($N$ = 12)), data are presented as mean (SD) and were analyzed by a one-way (B, C) or two-way (A, D) ANOVA with Tukey *post hoc* test, Kruskal–Wallis with Dunn's *post hoc* tests (E) or a one-way ANOVA + Welch's correction with Dunnett's post hoc test (F, statistical differences relative to Ctrl). ****$P$ < 0.0001. Exact $P$-values for all comparisons are listed in Appendix Table S1.

 © 2021 The Authors

 © 2021 The Authors

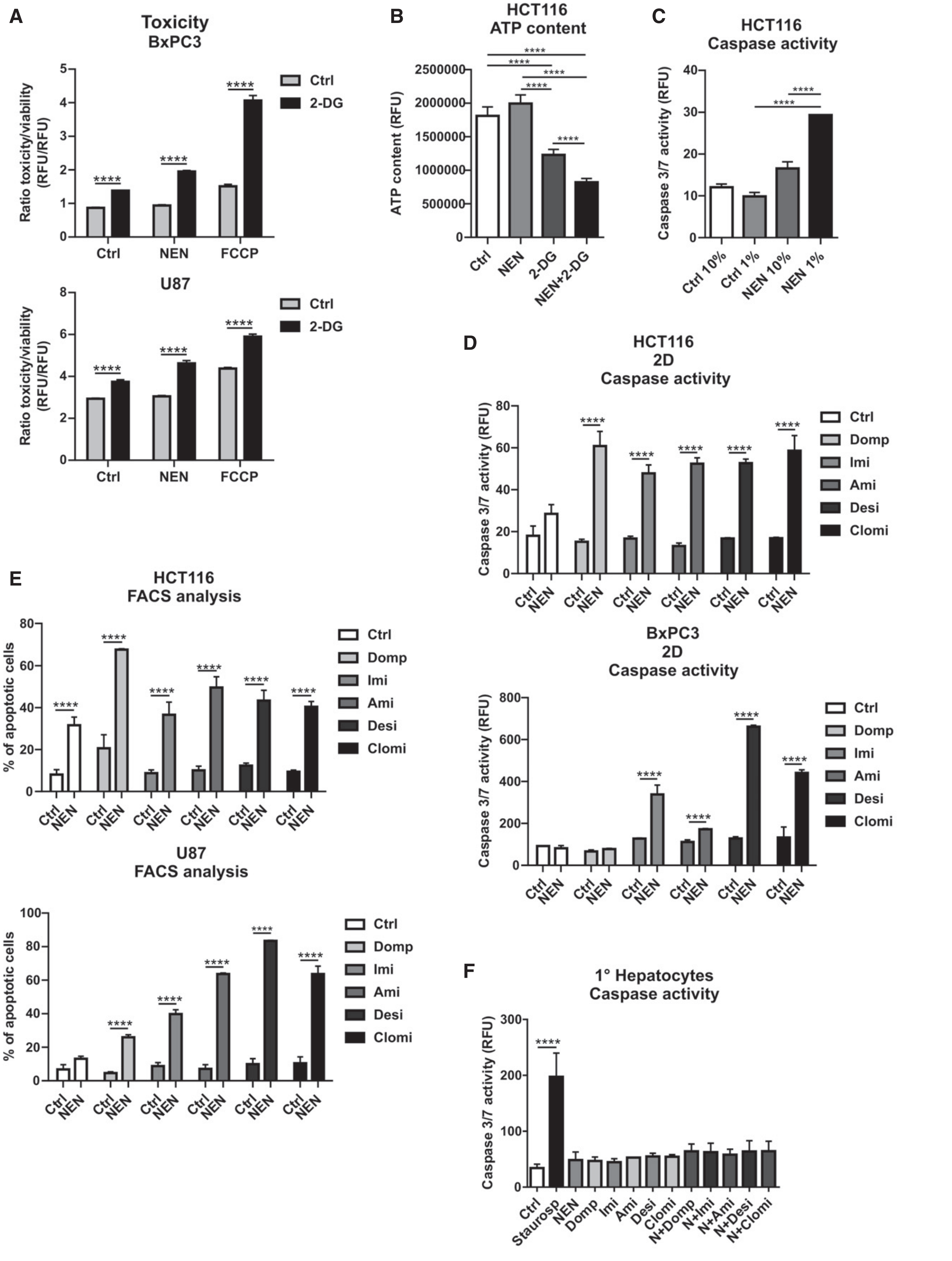
A
Toxicity
BxPC3
Ratio toxicity/viability (RFU/RFU)
Ctrl
2-DG
Ctrl NEN FCCP
U87
B
HCT116
ATP content
ATP content (RFU)
Ctrl NEN 2-DG NEN+2-DG
C
HCT116
Caspase activity
Caspase 3/7 activity (RFU)
Ctrl 10% Ctrl 1% NEN 10% NEN 1%
D
HCT116
2D
Caspase activity
Ctrl Domp Imi Ami Desi Clomi
BxPC3
2D
Caspase activity
E
HCT116
FACS analysis
% of apoptotic cells
U87
FACS analysis
F
1° Hepatocytes
Caspase activity
Ctrl Staurosp NEN Domp Imi Ami Desi Clomi N+Domp N+Imi N+Ami N+Desi N+Clomi
****

Figure EV1.

**Figure EV2. Induction of the ISR contributes to apoptosis in different cancer cell lines.**

A Relative Gadd34 mRNA expression levels in HCT116 cells treated as indicated for 16 h (NEN 1.2 μM, imipramine (Imi), desipramine (Desi), amitriptyline (Ami), each 30 μM, clomipramine (Clomi) 20 μM) ).

B Immunoblot analysis of CHOP, ATF4, phosphorylated (p-) and t-) eIF2alpha (eIF2a) of U87 cells treated as indicated for 24 h (NEN 1.2 μM; amitriptyline (Ami), each 30 μM).

C Densitometric quantifications of immunoblot analysis shown in Figs 2B and EV2B including CHOP, ATF4, phosphorylated (p-) and t-) eIF2alpha (eIF2a). HCT116 or U87 cells were treated as indicated for 16 or 24 h, respectively (NEN 1.2 μM; domperidone (Domp) amitriptyline (Ami), each 30 μM).

D Test of knockdown efficiency for siATF4 and siCHOP upon transfection of HCT116 cells, determined by qPCR.

E Caspase activity in BxPC3 cells grown as monolayer (2D) treated as indicated for 48 h (NEN 1.2 μM; domperidone (Domp), imipramine (Imi), desipramine (Desi), and amitriptyline (Ami), each 30 μM; clomipramine (Clomi) 20 μM, integrated stress response inhibitor (ISRIB) 1 μM).

F Ratio of toxicity and viability in U87 spheroids (3D) treated as indicated for 48 h (NEN 1.2 μM; domperidone (Domp), imipramine (Imi), desipramine (Desi), and amitriptyline (Ami), each 30 μM; clomipramine (Clomi) 20 μM, integrated stress response inhibitor (ISRIB) 1 μM).

Data information: Data are presented as mean (SD) and were analyzed by a one-way ANOVA with Dunnett's *post hoc* test (A ($N = 5$, besides NEN + Clomi ($N = 4$) and NEN + Desi ($N = 4$)), two-way ANOVA with Tukey *post hoc* test (C ($N = 3$), E ($N = 4$), F ($N = 4$) or Welsh's *t*-test (D ($N = 3$)). In (A), significance is indicated for the comparison of combinatorial treatments (NEN + TCAs) to controls. *$P < 0.05$; **$P < 0.01$; ***$P < 0.001$; ****$P < 0.0001$. Exact *P*-values for all comparisons are listed in Appendix Table S1.

Source data are available online for this figure.

 © 2021 The Authors

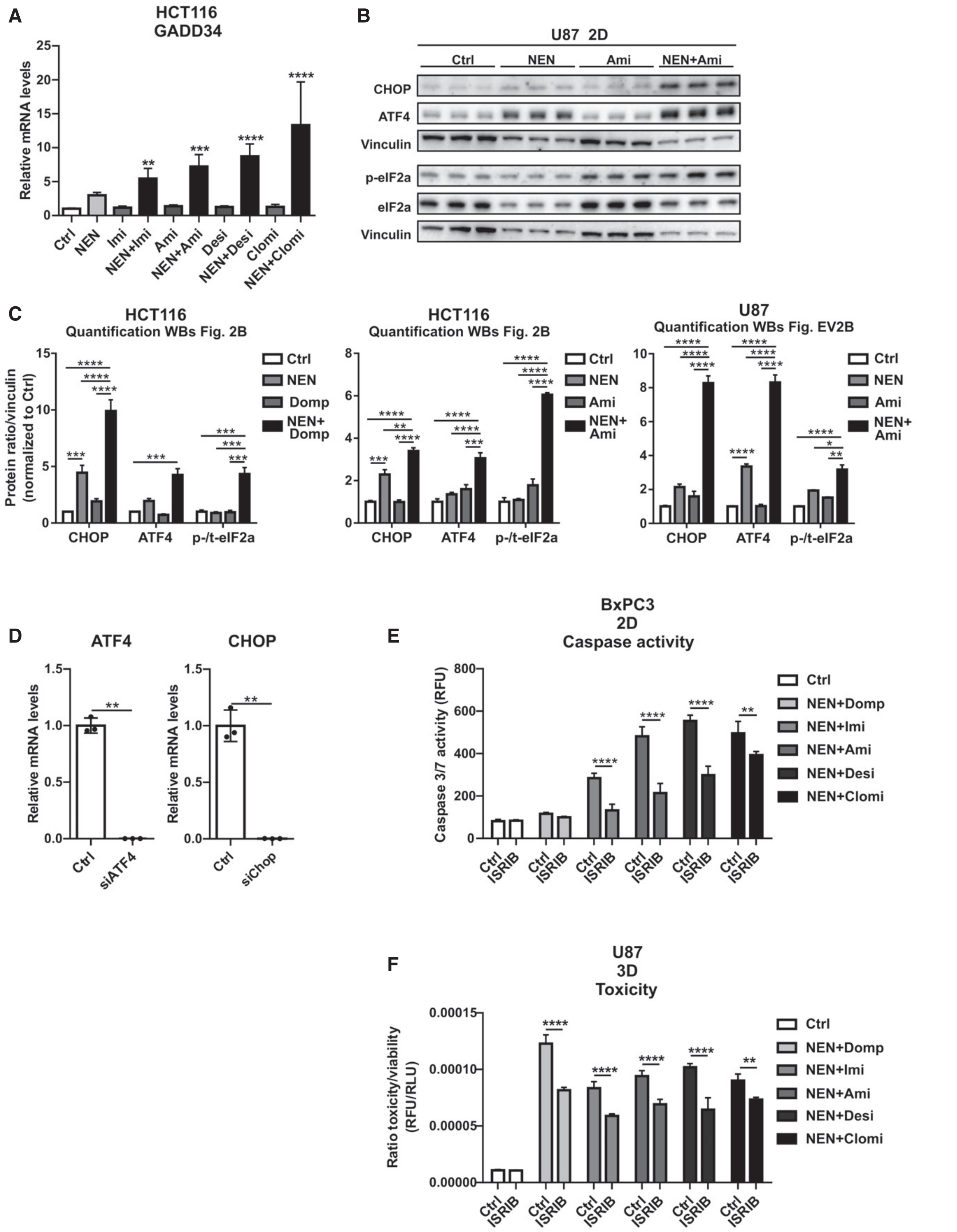
A
HCTI16
GADD34
Relative mRNA levels
Ctrl
NEN
Imi
NEN+Imi
Ami
NEN+Ami
Desi
NEN+Desi
Clomi
NEN+Clomi
B
U87 2D
Ctrl
NEN
Ami
NEN+Ami
CHOP
ATF4
Vinculin
p-eIF2a
eIF2a
Vinculin
C
HCT116
Quantification WBs Fig. 2B
Protein ratio/vinculin (normalized to Ctrl)
Ctrl
NEN
Domp
NEN+ Domp
CHOP
ATF4
p-/t-eIF2a
HCT116
Quantification WBs Fig. 2B
Ctrl
NEN
Ami
NEN+ Ami
U87
Quantification WBs Fig. EV2B
D
ATF4
CHOP
Relative mRNA levels
Ctrl
siATF4
siChop
E
BxPC3
2D
Caspase activity
Caspase 3/7 activity (RFU)
Ctrl
NEN+Domp
NEN+Imi
NEN+Ami
NEN+Desi
NEN+Clomi
ISRIB
F
U87
3D
Toxicity
Ratio toxicity/viability (RFU/RLU)

Figure EV2.

**Figure EV3. Combinatorial treatments induce the CLEAR network associated with a blockage in autophagy.**

A Relative mRNA expression levels of selected CLEAR network genes in HCT116 cells treated as indicated for 16 h (NEN (N) 1.2 μM, domperidone (Domp, D) 30 μM, imipramine (Imi), desipramine (Desi), amitriptyline (Ami), each 30 μM, clomipramine (Clomi) 20 μM).

B Immunoblot analysis of whole cell lysate (WL), cytoplasmic (Cyto) and nuclear (Nu) fractions of HCT116 cells using antibodies against the indicated proteins upon treatment of the cells as indicated for 16 h (Control (Ctrl), NEN (N) 1.2 μM, domperidone (D) 30 μM). GAPDH and acetyl-histone H3 (AC-H3) served as markers for cytoplasmic and nuclear fraction, respectively.

C Immunoblot analysis and quantification (mean ± SD) of LC3-II/LC3-I ratios and P62 in HCT116 cells. Cultured cells were treated with DMSO (Control), NEN or NEN and imipramine (Imi) as indicated (NEN, 1.2 μM; Imi, 30 μM) for 16 h. Protein band signal intensities were normalized to vinculin (loading control), and the ratios LC3-II bands LC3-I ($N = 3$) and p62 levels ($N = 3$) were analyzed by one-way ANOVA with Tukey *post hoc* test. *$P < 0.05$; **$P < 0.01$; ***$P < 0.001$; ****$P < 0.0001$.

D Immunoblot analysis and quantification (mean ± SD) of LC3-II/LC3-I ratios and P62 in HCT116 cells. Cultured cells were treated with DMSO (Control), NEN or NEN and amitriptyline (Ami) as indicated (NEN, 1.2 μM; Imi, 30 μM) for 16 h. Protein band signal intensities were normalized to vinculin (loading control), and the ratios LC3-II bands LC3-I ($N = 3$) were analyzed by one-way ANOVA with Tukey *post hoc* test. **$P < 0.01$; ***$P < 0.001$; ****$P < 0.0001$.

Data information: In (A), data are presented as mean (SD) and were analyzed by a one-way ANOVA with Tukey *post hoc* test. In (A), for the graphs with Imi, Ami, Desi and Clomi treatments ($N = 5$, besides NEN + Clomi ($N = 4$) and NEN + Desi ($N = 4$)), significance is indicated for the comparison of combinatorial treatments (NEN + individual TCAs) to controls. In (A), graphs with Domp (D) treatments ($N = 3$). *$P < 0.05$; **$P < 0.01$; ***$P < 0.001$; ****$P < 0.0001$. Exact *P*-values for all comparisons are listed in Appendix Table S1.

 © 2021 The Authors

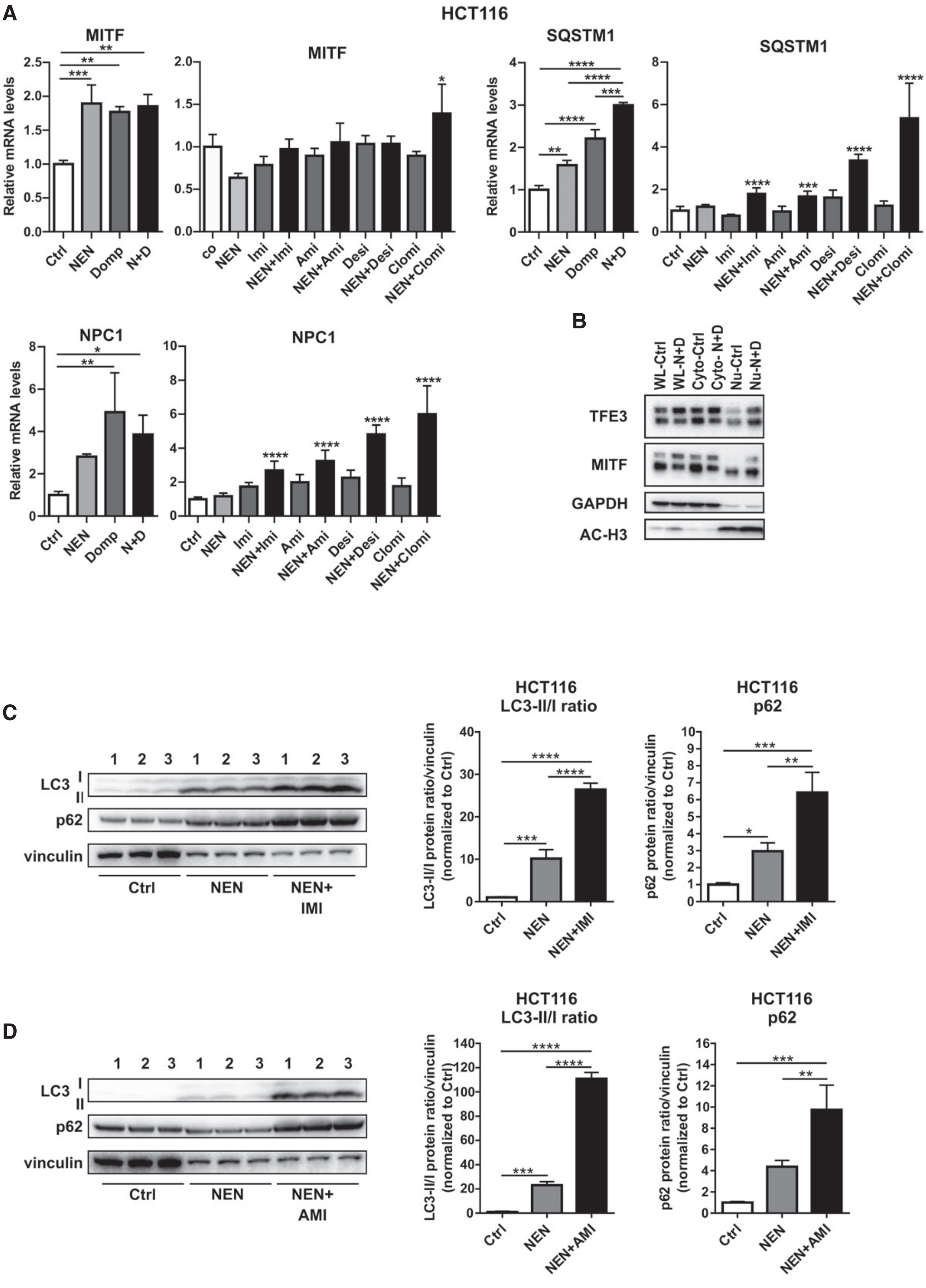
A
HCT116
MITF
SQSTM1
NPC1
Relative mRNA levels
Ctrl
NEN
Domp
N+D
co
Imi
NEN+Imi
Ami
NEN+Ami
Desi
NEN+Desi
Clomi
NEN+Clomi
B
WL-Ctrl
WL-N+D
Cyto-Ctrl
Cyto- N+D
Nu-Ctrl
Nu-N+D
TFE3
MITF
GAPDH
AC-H3
C
LC3 I
II
p62
vinculin
Ctrl
NEN
NEN+ IMI
HCT116 LC3-II/I ratio
LC3-II/I protein ratio/vinculin (normalized to Ctrl)
HCT116 p62
p62 protein ratio/vinculin (normalized to Ctrl)
NEN+IMI
D
NEN+ AMI
NEN+AMI

Figure EV3.

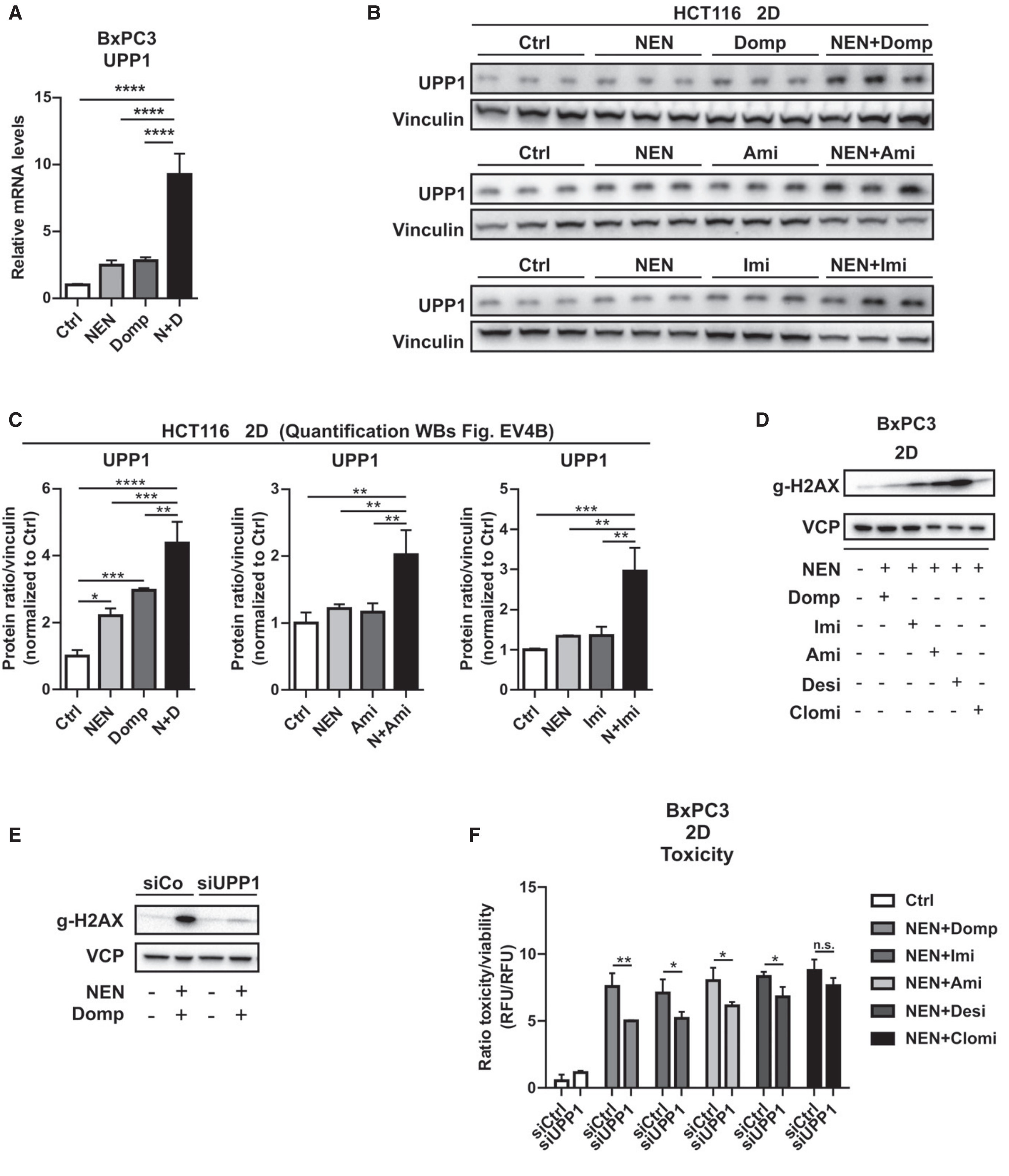


**Figure EV4.**


© 2021 The Authors

**Figure EV4. UPP1 induction contributes to drug toxicity in BxPC3 cells.**

- A Relative UPP1 mRNA expression levels in BxPC3 cells treated as indicated (NEN (N) 1.2 μM, domperidone (Domp, D) 30 μM).
- B Immunoblot analysis of UPP1 in HCT116 cells. Cultured cells were treated as indicated for 16 h (NEN, 1.2 μM; domperidone (Domp), imipramine (Imi), amitriptyline (Ami), each 30 μM).
- C Densitometric quantifications of immunoblot analysis of UPP1 shown in Fig EV4B (NEN (N), 1.2 μM; domperidone (Domp, D), imipramine (Imi), amitriptyline (Ami), each 30 μM). Protein band signal intensities were normalized to vinculin (loading control).
- D Immunoblot of whole cell lysates of BxPC3 cells using a gamma-H2AX-specific antibodies upon treatment of the cells as indicated for 18 h (NEN 1.2 μM; domperidone (Domp), imipramine (Imi), amitriptyline (Ami) and desipramine (Desi), each 30 μM; clomipramine (Clomi) 20 μM).
- E Immunoblot of BxPC3 whole cell lysates using antibodies against the indicated proteins upon treatment of the cells as indicated (NEN 1.2 μM, domperidone (Domp) 30 μM). Cells were transfected either with control (siCtrl) or UPP1-targeting (siUPP1) siRNAs 48 h prior to treatment.
- F Ratio of toxicity and viability of BxPC3 cells grown in monolayer (2D) and treated as indicated for 48 h (NEN 1.2 μM, domperidone (Domp), imipramine (Imi), desipramine (Desi), amitriptyline (Ami), each 30 μM, clomipramine (Clomi) 20 μM). Cells were transfected either with control (siCtrl) or UPP1-targeting (siUPP1) siRNAs 48 h prior to treatment.

Data information: In (A, C and F), data are presented as mean (SD) ($N = 3$) and were analyzed by a one-way ANOVA with Tukey *post hoc* test (A and C) or two-way ANOVA with Tukey *post hoc* test (F). *$P < 0.05$; **$P < 0.01$; ***$P < 0.001$; ****$P < 0.0001$. Exact *P*-values for all comparisons are listed in Appendix Table S1.

Source data are available online for this figure.

**Figure EV5. Drug combinations induce toxicity in patient-derived organoids and sensitize to Paclitaxel treatment.**

- A Ratio of toxicity and viability determined in pancreatic cancer-derived organoids from two patients ((left) PDO-42 and (right) PDO-48) treated as indicated for 3 (PDO-42) or 5 (PDO-48) days. Significant differences are shown for the comparison between drug treatments as indicated to the control condition (NEN (N) 1.2 μM or 2.5 μM, desipramine (D), each 30 μM, clomipramine (C) 20 μM, paclitaxel (Pacli), 2, 20 or 200 nM). As references for sensitization effects to standard chemotherapy, the bars showing single paclitaxel treatments using the indicated concentrations are highlighted in blue. Bars for controls (Ctrl), paclitaxel (Pacli), NEN (N) alone, and combined NEN + paclitaxel treatment are identical in the individual graphs (including respective graphs in Fig 7) for comparison to combinations with the respective TCA drugs as indicated.
- B Relative CHOP, UPP1 and PUMA mRNA expression levels in human pancreatic cancer-derived organoids (PDO-42) upon the indicated treatments determined by qPCR (NEN 1.2 μM; amitriptyline (Ami) 30 μM.

Data information: In (A), data are presented as mean (SD) ($N = 3$) and were analyzed by one-way ANOVA with Tukey *post hoc* test. In (B), data are presented as mean (SEM) ($N = 5$) and were analyzed by one-way ANOVA with Dunnett's post hoc test. *$P < 0.05$; **$P < 0.01$; ***$P < 0.001$; ****$P < 0.0001$. Exact *P*-values for all comparisons are listed in Appendix Table S1.

© 2021 The Authors

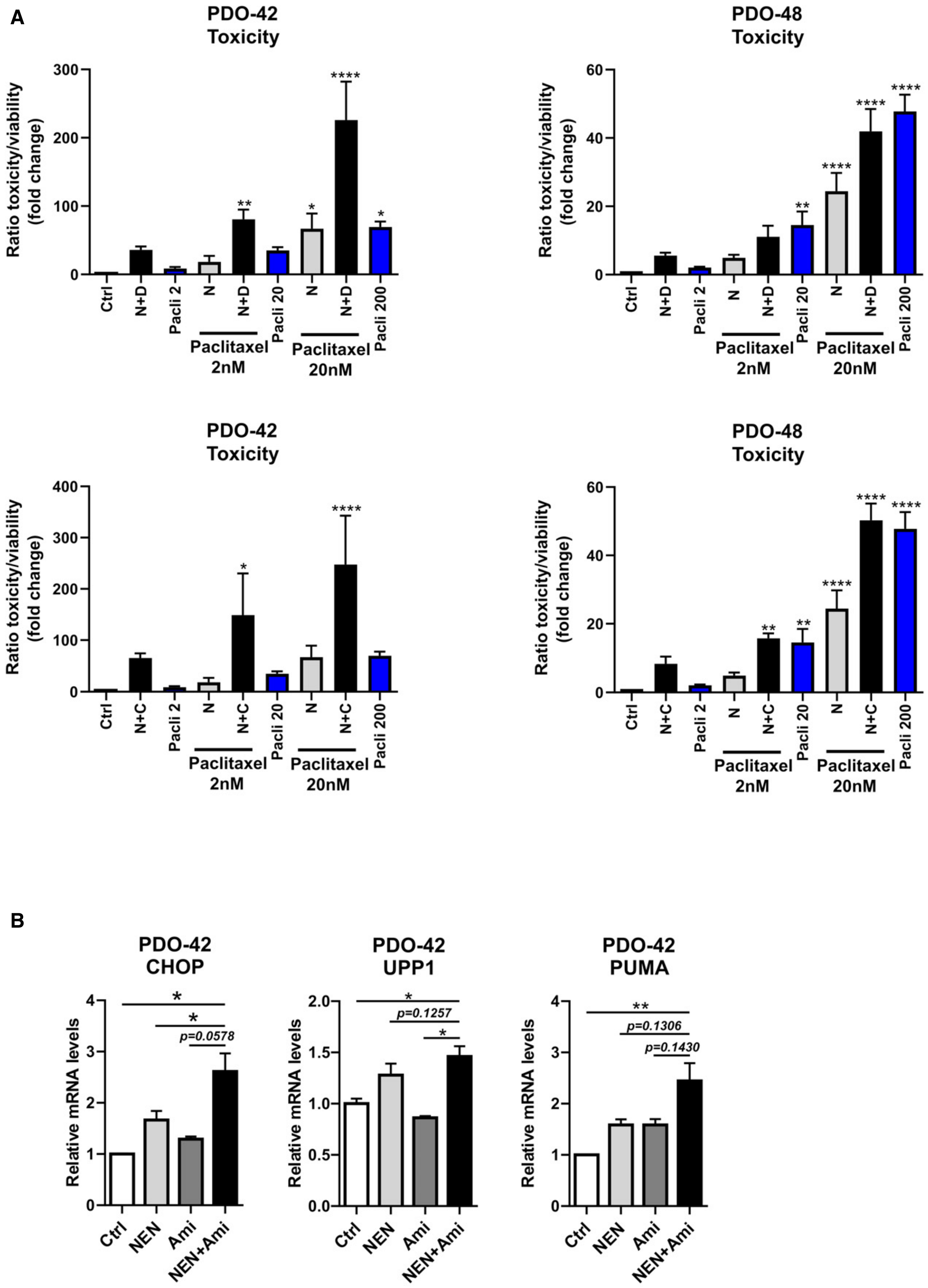


Figure EV5.

 © 2021 The Authors
