# Supplementary figures and images for: Combination therapies induce cancer cell death through the integrated stress response and disturbed pyrimidine metabolism
Source: EMBO Mol Med. 2021 Mar 5;13(4):e12461. doi: 10.15252/emmm.202012461 (PMC8033521; doi:10.15252/emmm.202012461)

Figure 2B

HCT116 cells

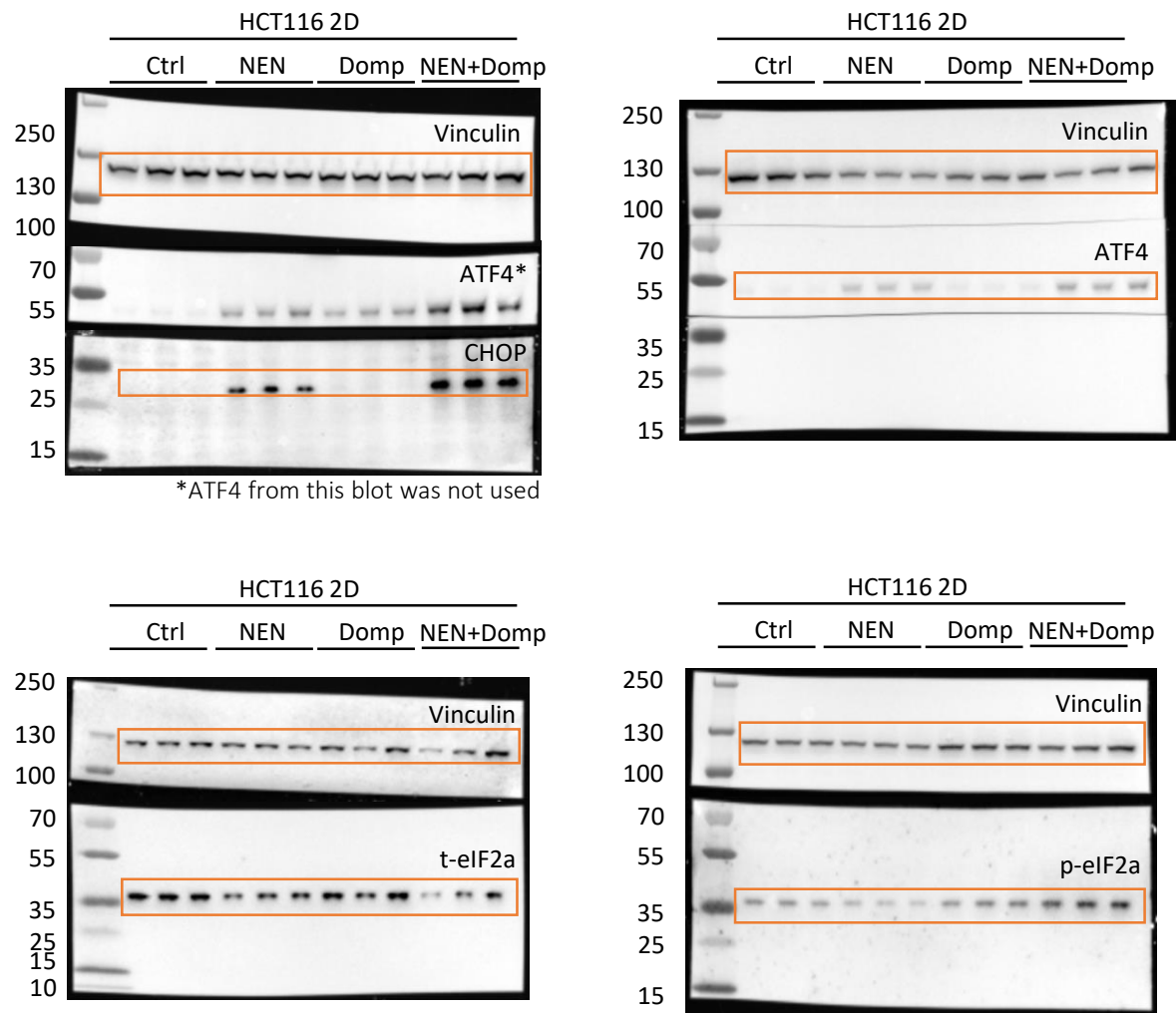

Figure 2B

HCT116 cells

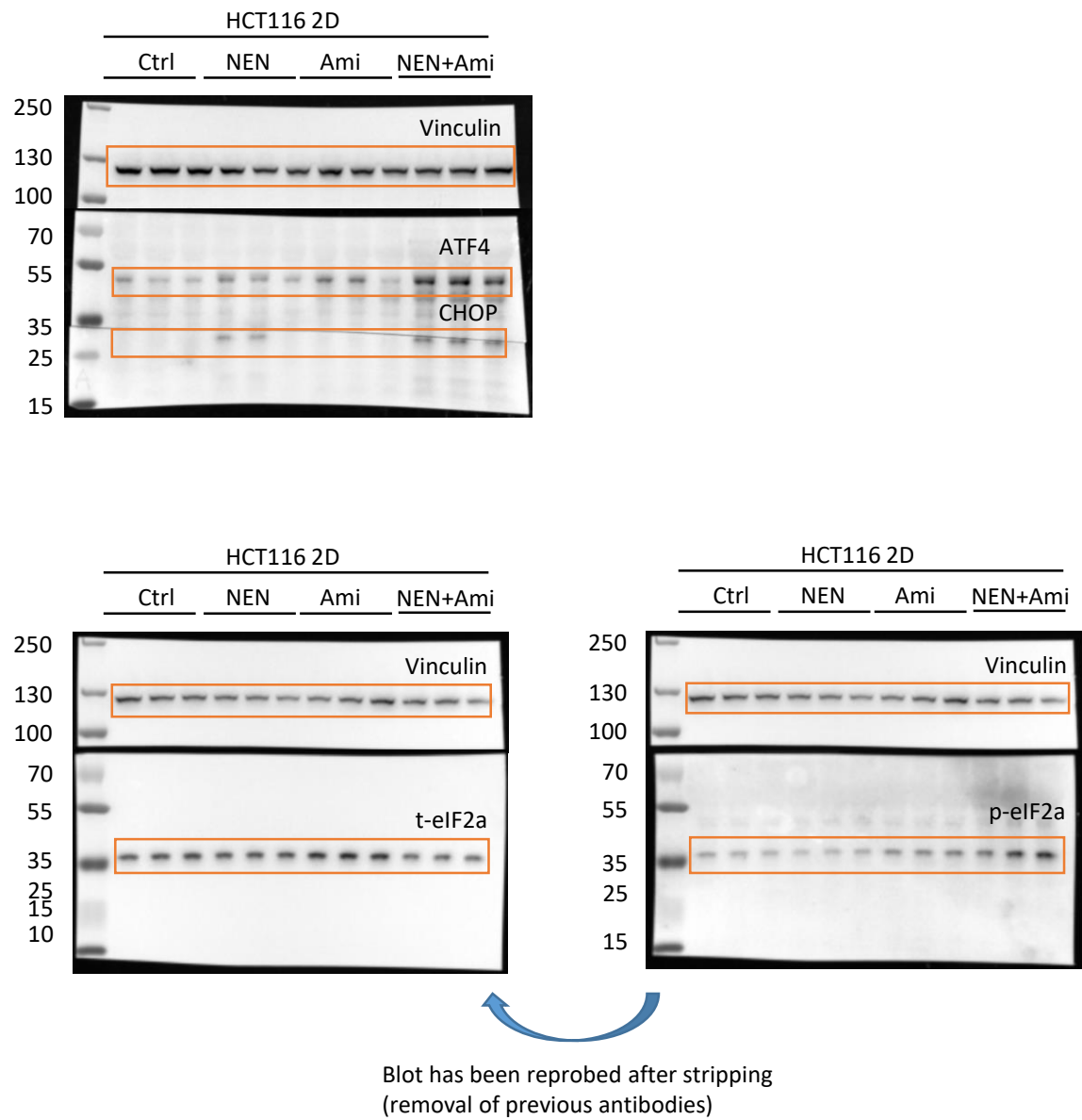

Supplement: Supplementary file 4 — Source Data for Figure 2 [file EMMM-13-e12461-s002.pdf]
