# Supplementary material for: Combination therapies induce cancer cell death through the integrated stress response and disturbed pyrimidine metabolism
Source: EMBO Mol Med. 2021 Mar 5;13(4):e12461. doi: 10.15252/emmm.202012461 (PMC8033521; doi:10.15252/emmm.202012461)

Source data – Hartleben et al.

Appendix Figure S8

HCT116 cells

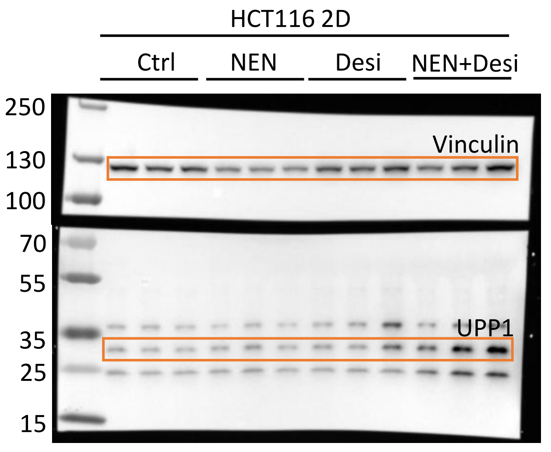

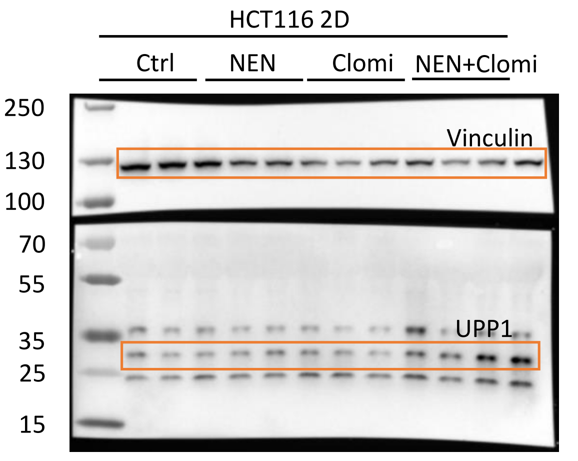

Source data

Appendix Figure S8

BxPC3 cells

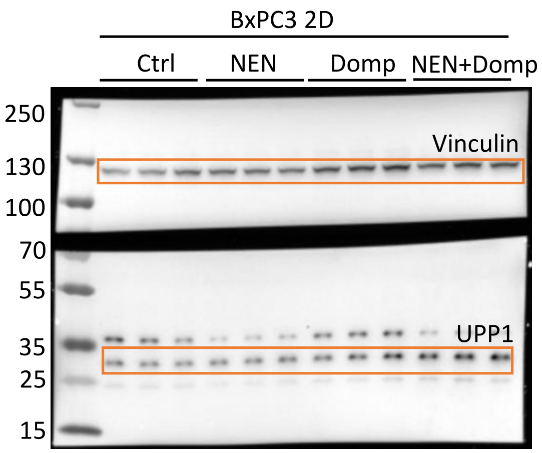

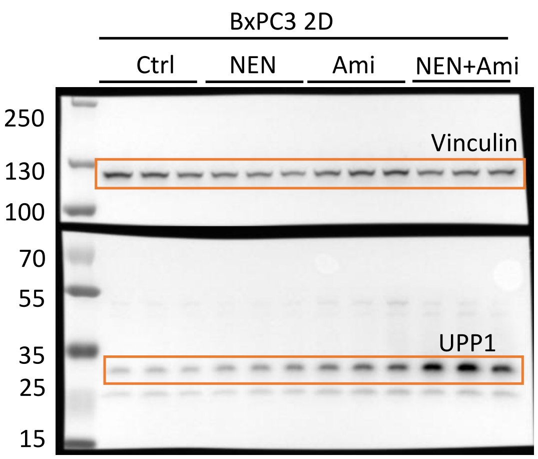

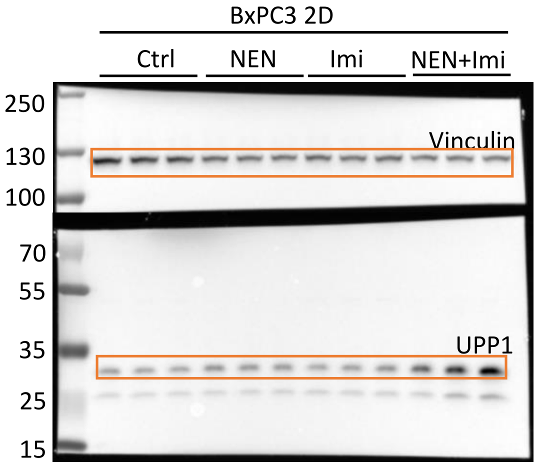

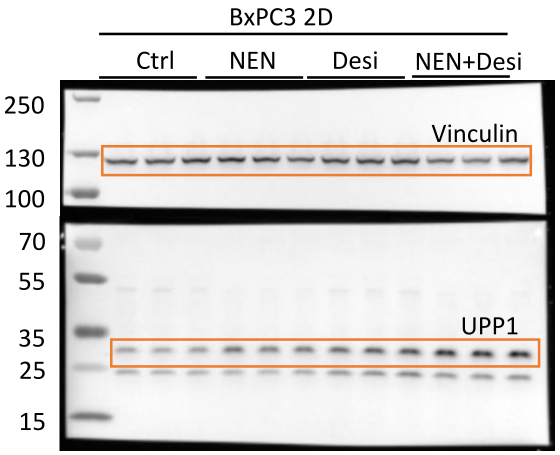

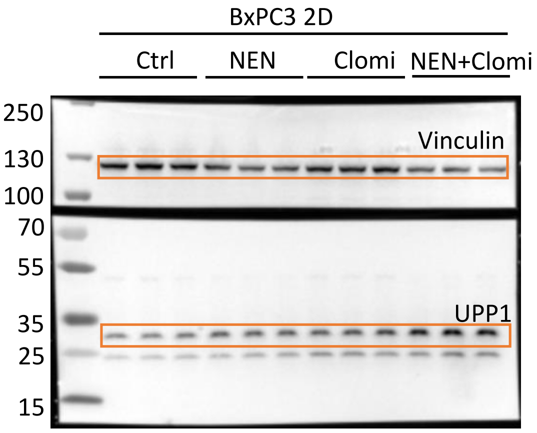

Source data

Appendix Figure S8

U87 cells

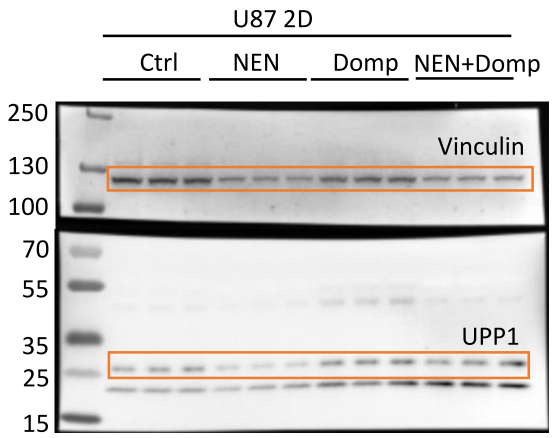

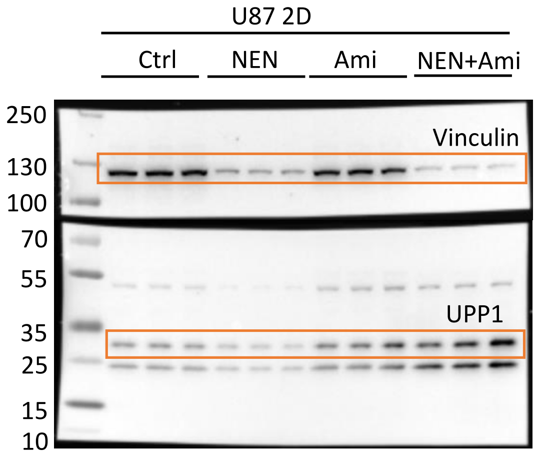

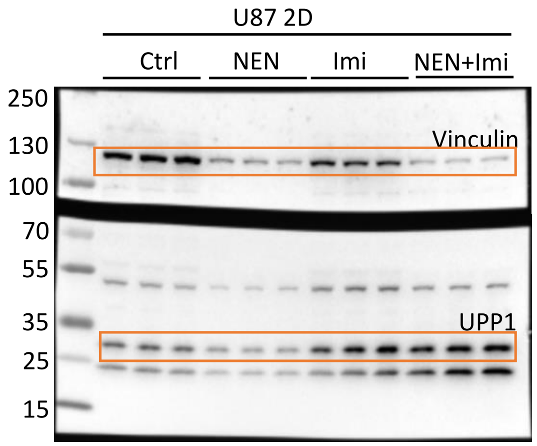

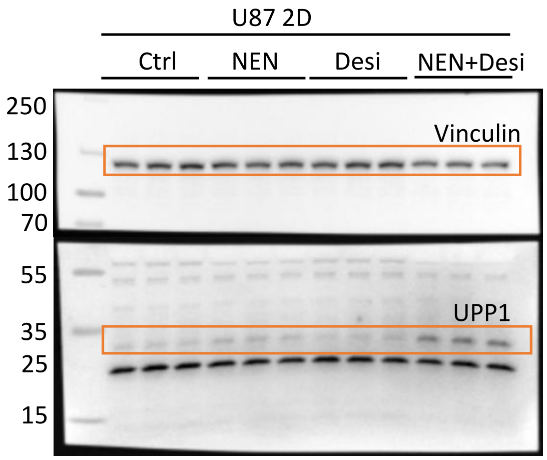

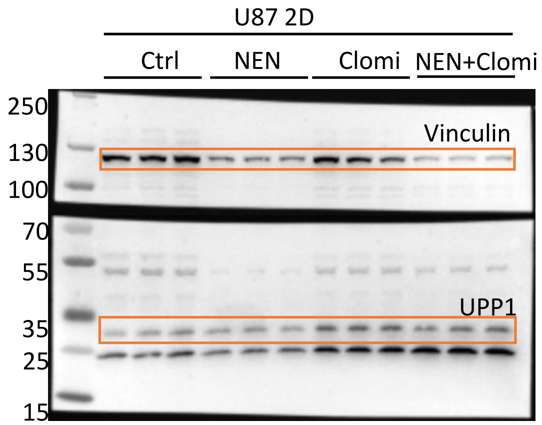

Supplement: Supplementary file 3 — Source Data for Expanded View and Appendix [file EMMM-13-e12461-s004.zip › Source_Data_for_Appendix_Figures.zip!/20210120_Source_data_for_Appendix_Figure_S8.pdf]
